# RNA-Binding Protein FXR1 Regulates p21 and TERC RNA to Bypass p53-Mediated Cellular Senescence in OSCC

**DOI:** 10.1371/journal.pgen.1006306

**Published:** 2016-09-08

**Authors:** Mrinmoyee Majumder, Reniqua House, Nallasivam Palanisamy, Shuo Qie, Terrence A. Day, David Neskey, J. Alan Diehl, Viswanathan Palanisamy

**Affiliations:** 1 Department of Oral Health Sciences and Center for Oral Health Research, College of Dental Medicine, Medical University of South Carolina, Charleston, South Carolina, United States of America; 2 Department of Urology, Henry Ford Health System, Vattikuti Urology Institute, Detroit, Michigan, United States of America; 3 Biochemistry and Molecular Biology, Medical University of South Carolina, Charleston, South Carolina, United States of America; 4 Department of Otolaryngology-Head and Neck Surgery, College of Medicine, Medical University of South Carolina, Charleston, South Carolina, United States of America; McGill University, CANADA

## Abstract

RNA-binding proteins (RBP) regulate numerous aspects of co- and post-transcriptional gene expression in cancer cells. Here, we demonstrate that RBP, fragile X-related protein 1 (FXR1), plays an essential role in cellular senescence by utilizing mRNA turnover pathway. We report that overexpressed FXR1 in head and neck squamous cell carcinoma targets (G-quadruplex (G4) RNA structure within) both mRNA encoding p21 (Cyclin-Dependent Kinase Inhibitor 1A (CDKN1A, Cip1) and the non-coding RNA Telomerase RNA Component (*TERC*), and regulates their turnover to avoid senescence. Silencing of FXR1 in cancer cells triggers the activation of Cyclin-Dependent Kinase Inhibitors, p53, increases DNA damage, and ultimately, cellular senescence. Overexpressed FXR1 binds and destabilizes *p21* mRNA, subsequently reduces p21 protein expression in oral cancer cells. In addition, FXR1 also binds and stabilizes TERC RNA and suppresses the cellular senescence possibly through telomerase activity. Finally, we report that FXR1-regulated senescence is irreversible and FXR1-depleted cells fail to form colonies to re-enter cellular proliferation. Collectively, FXR1 displays a novel mechanism of controlling the expression of p21 through p53-dependent manner to bypass cellular senescence in oral cancer cells.

## Author Summary

Understanding the mechanisms underlying evasion of cellular senescence in tumor cells is expected to provide better treatment outcomes. Here, we identify RNA-binding proteins FXR1 (Fragile X-Related protein 1), that is overexpressed in oral cancer tissues and cells bypasses cellular senescence through p53/p21-dependent manner. Once FXR1 is amplified in oral cancer cells, protein p21 is suppressed and non-coding RNA TERC expression is aided, resulting in reduction of cellular senescence and promotion of cancer growth. Here, we demonstrate the importance of FXR1 in antagonizing tumor cell senescence using human head and neck tumor tissues and multiple oral cancer cells including the cells expressing p53 wild-type and mutants. This finding is important as FXR1/TERC overexpression is associated with proliferation of HNSCC and poor prognosis, pointing to possible stratification of HNSCC patients for therapies.

## Introduction

Cellular senescence is a critical biological process occurring in normal and aging cells either due to developmentally programmed or DNA damage-induced causes. Cancer cells escape senescence by utilizing either transcriptional and/or co-transcriptional gene regulatory processes to control gene expression. For example, transcriptional activators including p53 [1,2] promote senescence by activating subset of genes and also get affected by upstream stress responses such as the DNA damage response (DDR). A majority of the transcriptionally activated genes such as p21 (CIP1/CDKN1A), p27 (CDKN1B), p16 (CDKN2A), and PTEN (Phosphatase and tensin homolog) are well-characterized for promoting cellular senescence through either activating p53 or p16-mediated senescence pathways [3]. Although changes in transcription play a major role in cellular senescence, the post-transcriptional changes associated with cellular senescence has not been well studied.

The post-transcriptional gene regulation is often controlled by RBPs in conjunction with noncoding RNAs [4]. Most importantly, aberrant expression of RBPs can alter the gene expression patterns and, subsequently, involve in carcinogenesis in multiple cancers including HNSCC [5]. A very few RBPs are known to be associated with senescence pathway by controlling mRNA processing, transport, stability, and translation of proteins responsible for senescence in mammalian cells. For example, RBPs like HuR, AUF1 and TTP can directly or indirectly control turnover and translation of mRNAs encoding senescence proteins [6,7,8]. In addition, the involvement of RBPs in DDR is rapidly growing and now they are considered as the major players in the prevention of genome instability [9]. RBPs prevent harmful RNA/DNA hybrids and are involved in DDR, and many different cell survival decisions. For example, in response to DNA damage, p53 induces RNPC1 expression and PCBP4 [poly(rC)-binding protein 4], which in turn represses translation of the mRNA encoding p53 and stability of the mRNA encoding p21, respectively [10,11]. Thus, RBPs are known to contribute to the cell fate decisions such as apoptosis and/or permanent cell cycle arrest to induce cellular senescence. A pro-senescence approach to cancer therapy is an attractive alternative approach to chemotherapeutic strategies [12]. However, abundant reports indicate that cellular senescence occurs in the pre-malignant stage of oral squamous cell carcinoma (OSCC) but is lost once malignant transformation has occurred [13,14,15,16,17]. In contrast, stress or oncogene-induced senescence (OIS) also reported in OSCC and indicated that OIS and its markers could play a role in OSCC tumor progression [18,19,20]. Furthermore, OSCC cells expressing high-risk p53 mutations are sensitized to cisplatin therapy by the selective wee-1 kinase inhibitor, MK-1775, which subsequently promoted mitotic arrest and cellular senescence [21]. Thus, understanding the molecular mechanisms that underpin RBP-mediated senescence may yield invaluable data for the management of OSCC.

FXR1 belongs to the Fragile X-Related (FXR) family of RBPs, which also includes Fragile X Mental Retardation 1 (FMRP) and Fragile X-Related 2 (FXR2). FXR1 is frequently amplified in chromosome 3q26-27 in lung squamous cell carcinomas [22]. A recent observation indicates that FXR1 is a key regulator of tumor progression and is critical for growth of non-small cell lung cancer cell (NSCLC), and head and neck squamous cell carcinoma (HNSCC) [23]. Similar to the functions of other RBPs, FXR1 is involved in mRNA transport, translational control, and mRNA binding via AU-rich elements (ARE) or G4 RNA structures [24,25]. FXR1 is shown to bind to G4-RNA structure at the 3’-UTR of *p21* and reduce its half-life in mouse C2C12 cells [26]. The G4 RNA structure containing human telomerase reverse transcriptase (hTERT) and its RNA component *TERC RNA* [27], are suppressors of cellular senescence in a variety of cells as deregulation of their function leads to the progressive shortening of telomere [28]. The regulatory mechanisms controlled by RNA G4 structures involve the binding of protein factors that modulate G4 RNAs turnover and serve as a bridge to recruit additional protein regulators. For example, G4 structure forming sequences protect *TERC* from degradation and interact with RNA helicase associated with AU-rich element (RHAU), a DEAH-box RNA helicase that exhibits G quadruplex-RNA binding and resolving activity [29]. In this report, we have identified that FXR1, overexpressed in oral squamous cell carcinoma, binds and destabilizes G4 containing RNA *p21* and in turn reduces its protein expression in oral cancer cells. Thus controls cell cycle at G0/G1 phase, maintains cancer cell proliferation and evades cellular senescence. In addition, FXR1 associates and stabilizes non-coding RNA *TERC* resulting in suppression of cellular senescence and increased cancer growth. Thus, FXR1 is an important protein that regulates RNAs such as *p21* and *TERC* to promote cancer progression.

## Results

### FXR1 is overexpressed in HNSCC

**Fig 1:**
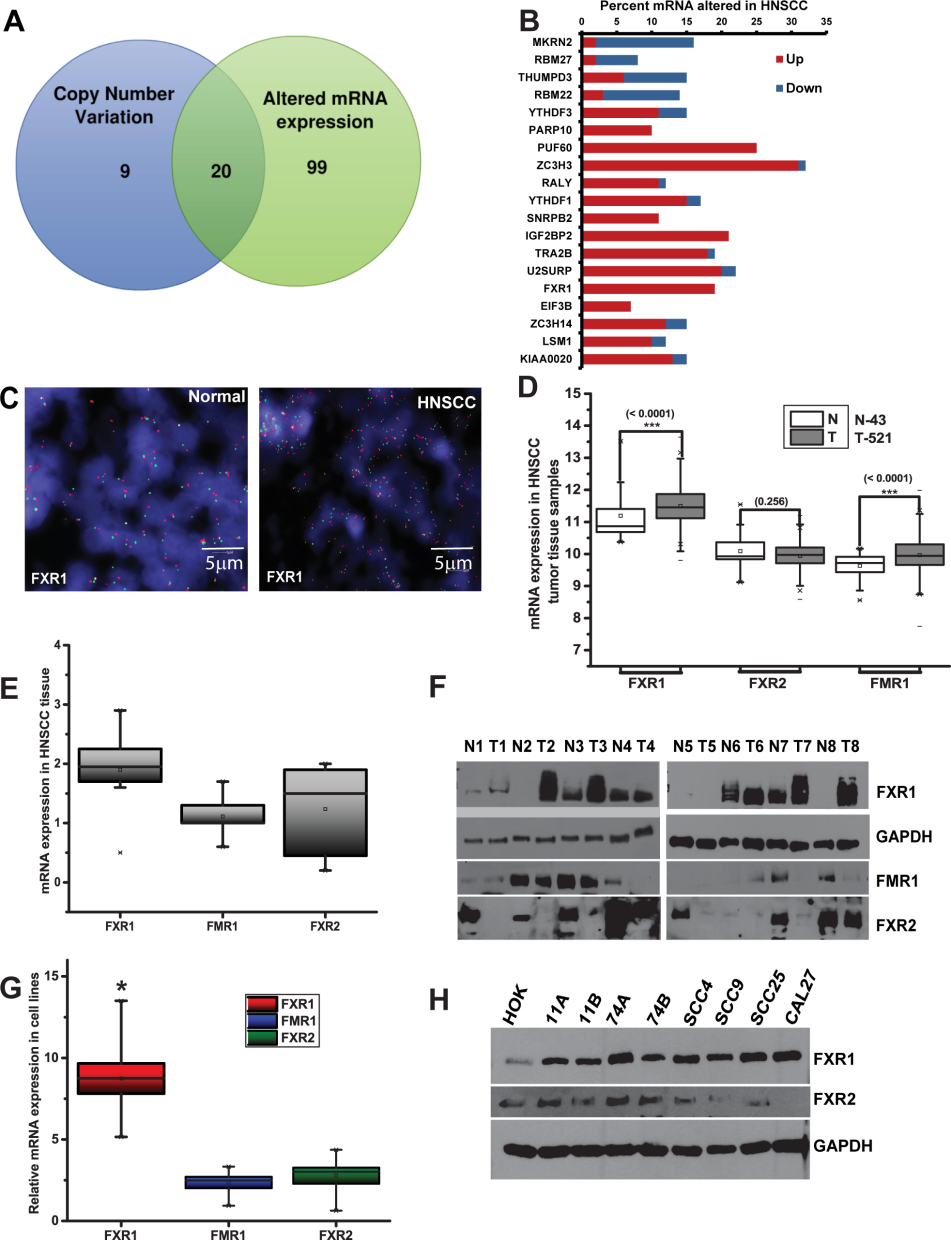
FXR1 is overexpressed in head and neck squamous cell carcinoma. (A) Comparison of altered copy number and mRNA expression level of RBPs in TCGA HNSCC data sets. (B) Percent mRNA expression level of top altered RBPs in 279 TCGA HNSCC tumor samples. Frequencies of expression levels are shown as a percentage of all cases. (C) Representative dual color FISH of *FXR1* gene (green spots) and a chromosome 3 loci probe (red spots) in HNSCC (scale bar 5μm). (D) FXR family of proteins (FXR1, FXR2 and FMR1) coding mRNA expression levels in TCGA database where cancer genome browser was used to visualize the data sets. N = Normal; and T = Tumor. (E) qRT-PCR estimate of mRNA levels of FXR proteins in eight matched HNSCC tumor compared to normal adjacent tissue samples. (F) Immunoblot analysis of eight HNSCC and matched adjacent normal tissue for FXR1, FXR2, FMR1, and GAPDH. (G) qRT-PCR analysis of mRNA levels of FXR1, FXR2 and FMR1 in eight HNSCC cell lines compared to HOK. (H) Immunoblot analysis of FXR1, FXR2, and FMR1 in HNSCC cell lines compared to HOK. GAPDH serves as a loading control.

To determine whether expression of RBPs is different in HNSCC, we utilized the cbioportal cancer genomics database (www.cbioportal.org) to examine the Cancer Gene Atlas (TCGA) in Head and Neck cancer study. We first analyzed copy number variation of the predicted and well-conserved 424 RBPs [30]. The TCGA HNSCC dataset contains tumor samples from 516 patients, of which 302 were analyzed for copy number alterations and mutation status with 5% cut off (Fig 1A, S1 Table). Next, to test the mRNA expression pattern of 424 RBPs, we set an mRNA expression onset of two standard deviations above or below the mean z-score, to identify patients with highly altered RBP mRNA levels. Using an arbitrary cut-off (RBP EXP > = 1.5), we identified 123 RBPs which were altered in 10% (51/516) or more of HNSCC patients (Fig 1B, S2 Table). Among those shown significant alteration, FXR1 showed a 31% alteration in a combined DNA copy number and mRNA in 279 tumor samples. Moreover, patients without FXR1 mRNA alteration showed a significant (*p*<0.01) overall survival rate compared to the ones with mRNA alteration, by Kaplan- Meier estimates (S1A Fig). DNA copy number status of FXR1 was independently verified by fluorescence in situ hybridization (FISH) analysis in a HNSCC tissue microarray (TMA). As shown in Fig 1C (S3 Table), FXR1 is highly expressed in tumor compared to normal adjacent tissues. Next, we used UCSC Cancer Genome Browser to compare the mRNA expression levels of tumor and normal tissues in a large cohort of patients (Normal = 43 and Tumor = 521). FXR1 mRNA levels along with two other FXR1 families of proteins FMR1 and FXR2 were determined in 521 tumor samples. As shown in Fig 1D (S4 Table), FXR1 and FMR1 mRNAs are significantly (*p*<0.0001) expressed in tumor samples compared to normal. However, we did not observe a significant difference in expression for FXR2 in normal adjacent and HNSCC tumor tissues. Next, we tested the mRNA levels of FXR1 in eight matched tumor vs normal adjacent samples (obtained from HNSCC patients from MUSC biorepository) by qRT-PCR (clinical parameters are tabulated in S5 Table). As shown in Fig 1E, FXR1 mRNA is overexpressed in tumor compared to normal adjacent tissues, whereas FMR1 and FXR2 mRNA levels are comparable to their normal mRNA expression. All the values were normalized to 1 corresponding to their normal adjacent tissues. To confirm the above observations, the levels of FXR1, FMR1, and FXR2 proteins from eight representative matched HNSCC tumor and normal adjacent samples were analyzed. As expected the level of FXR1 is highly amplified in cancer tissues compared to normal adjacent tissues and we do not see differential expression of FMR1 and FXR2 (Fig 1F). Next, we tested the mRNA levels of FXR1, FXR2 and FMR1 in eight HNSCC cell lines compared to the normal primary human oral keratinocytes (HOK, value was taken as 1). As shown in Fig 1G, FXR1 mRNA is significantly (*p*<0.05) overexpressed in HNSCC cells compared to HOK cells, whereas FMR1 and FXR2 mRNAs are not significantly overexpressed. Finally, the protein levels for FXR1, FXR2 and FMR1 were determined in HNSCC cell lines compared to HOK. FXR1 protein expression is high in all the cell lines tested compared to HOK (Fig 1H). Unlike the mRNA expression pattern, FXR2 does not uniformly express in cell lines (Fig 1H). The FMR1 protein is not detected in these cell lines. Collectively, our data show that FXR1 DNA, mRNA, and protein is amplified and expressed at high levels in HNSCC tumor tissues and cell lines.

### Overexpressed FXR1 subdues cellular senescence through cell cycle arrest and DNA damage

**Fig 2:**
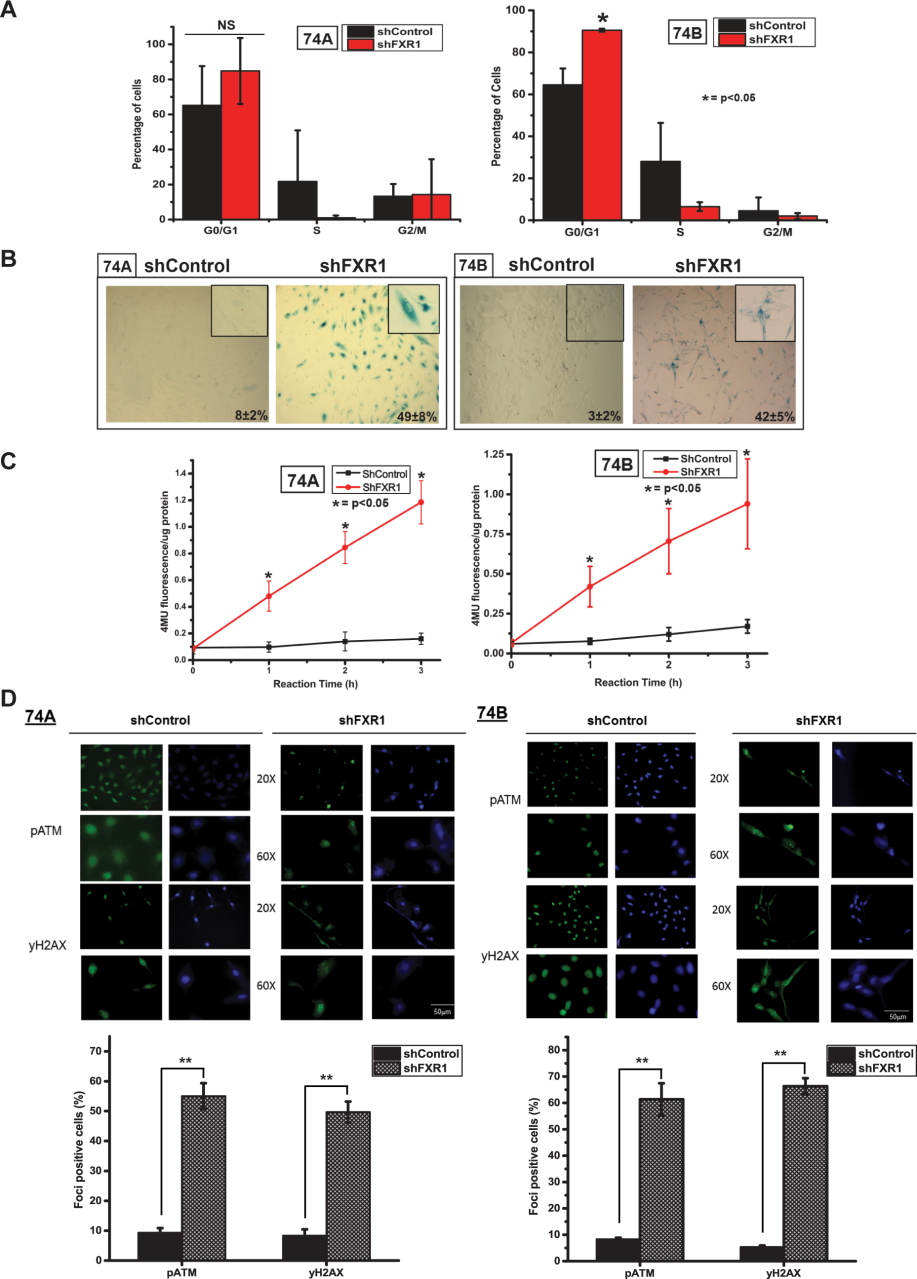
Silencing of FXR1 in cancer cells promotes cellular senescence. (A) After 72hrs of shRNA treatment, UMSCC74A and 74B FXR1 KD and control cells are fixed and stained with propidium Iodide (PI), and analyzed by fluorescence-activated cell sorting to evaluate the number of cells in different stages of cell cycles. (B) 72 hours post-transduction staining for SA-β-gal in FXR1 depleted UMSCC74A and 74B cells. Three independent fields for each experiment are used here for quantification. (C) Measurement of *MUG* conversion to 4-MU by senescence associated β-galactosidase in FXR1 depleted UMSCC74A and 74B cells. (D) Representative immunofluorescence images of pATM and γ-H2AX staining FXR1 depleted UMSCC74A and 74B cells compared to control. Quantification of foci containing cells is given under each cell lines. (*p<0.05, **p<0.005, ***p<0.0005).

We have utilized two different shRNA constructs and both shRNAs are able to silence FXR1 protein in HNSCC cells (S1B Fig). We selected shRNA-2 (TRCN0000159153) for further gene silencing experiments in this report. However, in order to delineate the off target effects of shRNAs, we used additional shRNA-1 (TRCN0000158932) to ascertain the important experiments (S1C, S1D and S1E Fig). First, to test whether knock down (KD) of FXR1 has any effect on cell viability, we used MTT (3-(4, 5-dimethylthiazol-2-yl)-2, 5-diphenyltetrazolium bromide) colorimetric assay. As shown in S1F Fig, a significant decrease in number of viable cells is observed in both UMSCC74A (*p*<0.05) and UMSCC74B (*p*<0.01) cells with FXR1 KD compared to control, indicating FXR1 plays a key role in oral cancer cell viability and/or survival. To investigate the underlying mechanism of reduction in number of viable cells following FXR1 KD, we performed cell cycle analysis of the FXR1 KD and control cells by flow cytometry. As shown in Fig 2A, FXR1 KD induces cell cycle arrest in G0/G1 providing evidence that FXR1 might regulate cell division in oral cancer. Interestingly, FXR1 KD does not induce apoptosis in UMSCC74A and UMSCC74B cells, as demonstrated by the absence of cleaved PARP and Caspase-3 (S1G Fig). Cell cycle arrest is one of the key features of cellular senescence [31]. Hence, to test whether FXR1 KD-induced cell cycle arrest is associated with induction of cellular senescence, we assessed the expression of senescence-associated β-galactosidase (SA-β-gal) activity. As shown in Fig 2B, FXR1 KD UMSCC74A and UMSCC74B exhibit increased SA-β-gal staining compared to control cells. In addition, silencing of FXR1 in multiple oral cancer cells also promotes senescence by positive SA-β-gal staining (S2A Fig). Furthermore, to quantify the β-gal enzyme activity, we measured *MUG* (4-Methylumbelliferyl β-D-Galactopyranoside) conversion by senescence associated β-galactosidase to 4-MU as described by [32]. As shown in Fig 2C, both FXR1 KD UMSCC74A and UMSCC74B cells, the 4MU fluorescence per microgram is significantly high (*p<*0.05) compared to control shRNA treated cells. To confirm that cells underwent DNA damage associated cellular senescence, we used immunofluorescence to study DNA damage. An important step during the cellular response to DNA double stand break (DSB) is the phosphorylation of histone H2AX at the break site, giving rise to discrete nuclear foci, termed γ-H2AX foci and also phospho-ATM (pATM) foci, accumulation at the break sites and can also be visualized as distinct foci [33]. Here, we silenced FXR1 and estimated the level of γ-H2AX and pATM foci in both oral cancer cell lines. As shown in Fig 2D, the appearance of spontaneous γ-H2AX and pATM foci in FXR1 depleted cells demonstrate that DSBs occur after silencing of FXR1 compared to control shRNA treated cells. Representative quantitative information of the foci formation is shown under each cell line. Cells containing two or more foci are counted as positive [34]. Next, to confirm that depletion of FXR1 promotes cellular senescence; we have used FXR1 knockout mouse embryonic fibroblasts (MEFs) for SA-β-gal qualitative assay. As expected, FXR1 KO MEFs showed bright staining of SA-β-gal compared to WT MEFs (S2B Fig). Collectively, our analyses indicate that silencing of FXR1 in oral cancer cells led to DNA damage, cell cycle arrest and cellular senescence.

### FXR1 KD induces senescence by activating cell cycle regulatory proteins

**Fig 3:**
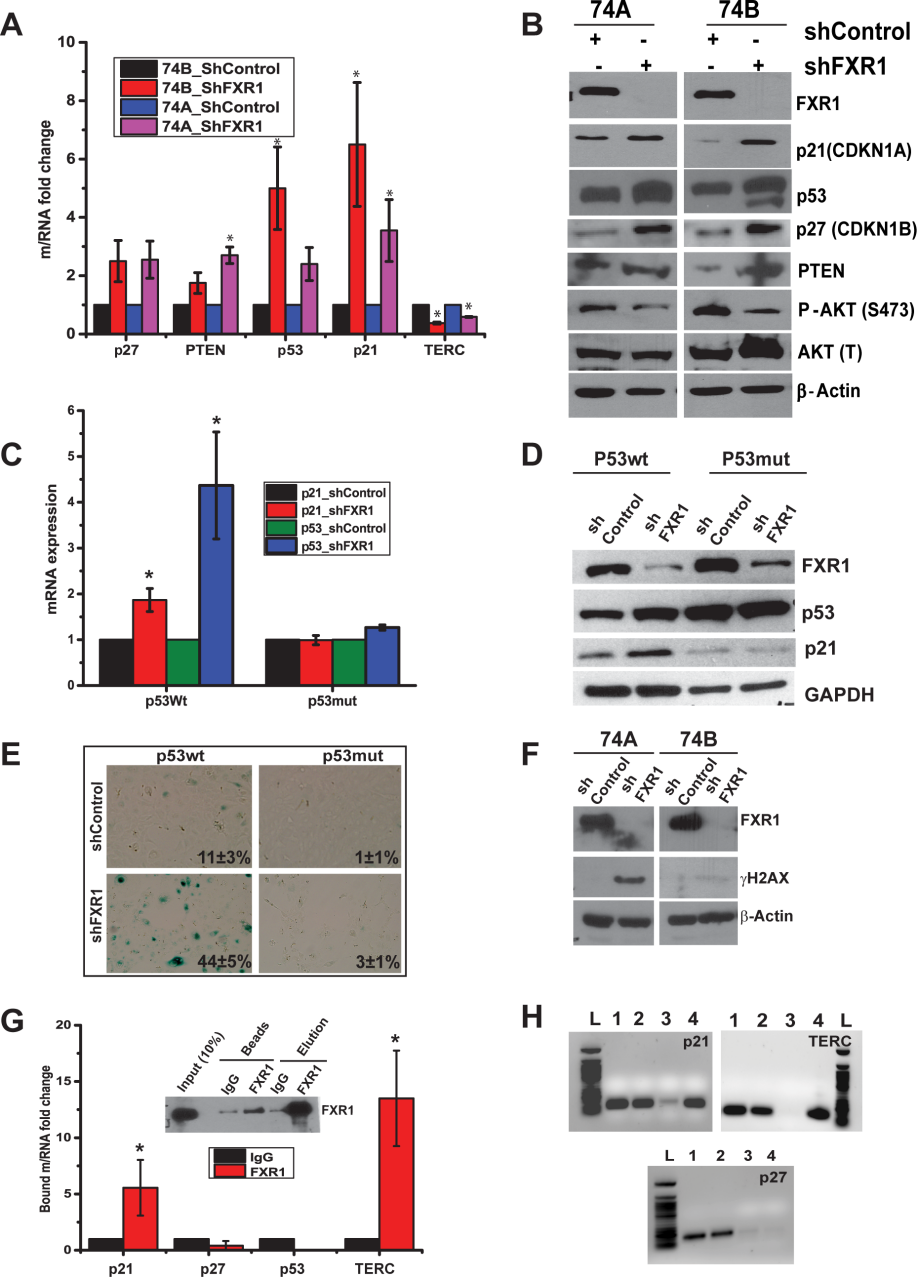
Depletion of FXR1 alters cell cycle proteins in HNSCC. (A) Relative quantity of RNAs extracted from control and FXR1 KD cells are estimated by using qRT-PCR. GAPDH serves as a control. (B) Immunoblot analysis of senescence marker proteins in both FXR1 depleted UMSCC74 and 74B cells. β-Actin used as a loading control. (C) Relative quantity of p21 and p53 mRNAs, extracted from shcontrol and shFXR1 treated P53wt and P53mut cells, are estimated by using qRT-PCR. GAPDH serves as a control. (D) Western blot analysis of FXR1 depleted p53Wt and mutant oral cancer cells. GAPDH is used as a loading control. (E) FXR1 depleted p53Wt and mutant oral cancer cells stained with SA-β-gal. (F) Western blot yH2AX expression in UMSCC74A and UMSCC74B cells upon FXR1 KD. (G) Protein lysates of UMSCC74B cells are subjected to RNP IP followed by qRT-PCR analysis to measure the relative quantities of RNAs in FXR1 IP compared with control IgG IP. GAPDH serves as a loading control. (H) RT-PCR of products acquired from inputs and IPs respectively from Mouse IgG as control (Lanes 1, 3) and IP of FXR1 (Lanes 2, 4) were separated and visualized by agarose gel electrophoresis. It clearly shows that p21 mRNA and TERC RNA are enriched in the IP samples, whereas p27 is not. NEB 50bp DNA ladder #N3236S (L) was loaded as a molecular marker. (*p<0.05, **p<0.005, ***p<0.0005).

To test if FXR1 regulated cellular senescence occurs through alteration of post-transcriptional gene expression; we analyzed gene expression of senescence markers. As shown in Fig 3A, qRT-PCR analysis of *p21*, *p27*, *p53*, *and PTEN* revealed that their mRNA levels have increased in the absence of FXR1 in both UMSCC74A and UMSCC74B cells. Intriguingly, the level of *TERC RNA* has decreased significantly (*p<*0.05) in the absence of FXR1. Consistent with this analysis the protein levels of p53, PTEN, p21, and p27 (CDKN1B) increased upon FXR1 KD in UMSCC74A and UMSCC74B cells (Fig 3B). In addition, reduced levels of pAkt (Ser-473) is observed in FXR1KD UMSCC74A and UMSCC74B cells compared with control and total Akt (Fig 3B), indicates that FXR1 regulated cellular senescence is possibly aided through inactivation of phosphatidylinositol 3 kinase/Akt signaling pathway. As we observe an increase in p53 protein upon FXR1 KD (Fig 3B), next we tested whether p53 plays a role in promoting senescence in the absence of FXR1. Many human tumors are not entirely lacking p53, but instead they have “hot spot” p53 mutations which serve as tumor suppressors. Here, we used two isogenic oral cancer cell lines in the background of PCI13 where they express wild type (Wt) and mutant p53 (C238F), respectively, as described [35]. Fig 3C illustrates the levels of *p21* and *p53* mRNAs upon FXR1 KD. Both mRNAs exhibit a significant increase in p53Wt cells in comparison with p53 mutant cells. To test the mRNA changes alter the expression of proteins, we tested the protein expression patterns of p53 and p21 in FXR1 KD cells. Interestingly, upregulation of p53 and p21 protein levels are observed in the absence of FXR1 in WT p53 cell line. Moreover, silencing of FXR1 in WT p53 cells exhibit SA-β-gal staining compared to the mutant (Fig 3E). This observation demonstrates that FXR1-regulated senescence utilizes p53 activated senescence pathway in oral cancer cells. Next, to corroborate our observation in Fig 2D that silencing of FXR1 promotes DNA damage; we determined the H2AX expression (phosphorylation of H2AX at Ser 139 (γ-H2AX) correlates well with each double stranded DNA break) under FXR1 KD condition. As shown in Fig 3F, FXR1 KD UMSCC74A and 74B cells express γ-H2AX compared to control indicating that DNA damage occurs in the absence of FXR1. Next, to determine whether the senescent protein coding mRNAs are associated with FXR1 to exert their function, we employed RNP IP assay as described [36]. As shown in Fig 3G, both *p21* and *TERC* are associated with FXR1 in comparison with *p27* and *p53*. FXR1 IP data in the figure is added to show that FXR1 efficiently binds to the beads and elutes out with the bound mRNAs under RNP IP conditions. Interestingly, both the 3’-UTR of *p21* and full-length *TERC* RNAs contain G4 RNA sequences (S2D Fig), identified by using QGRS mapper software [37]. Agarose gel analyses of PCR amplified RNAs obtained from FXR1 RNA IP samples (Fig 3G) show that *p21* and *TERC* bind to FXR1 protein (Fig 3H). Altogether, these studies indicate that FXR1 coordinates the expression of several senescence-associated genes; most importantly it binds and regulates the expression of both *p21* and *TERC* in oral cancer cells.

### FXR1 destabilizes *p21* mRNA

**Fig 4:**
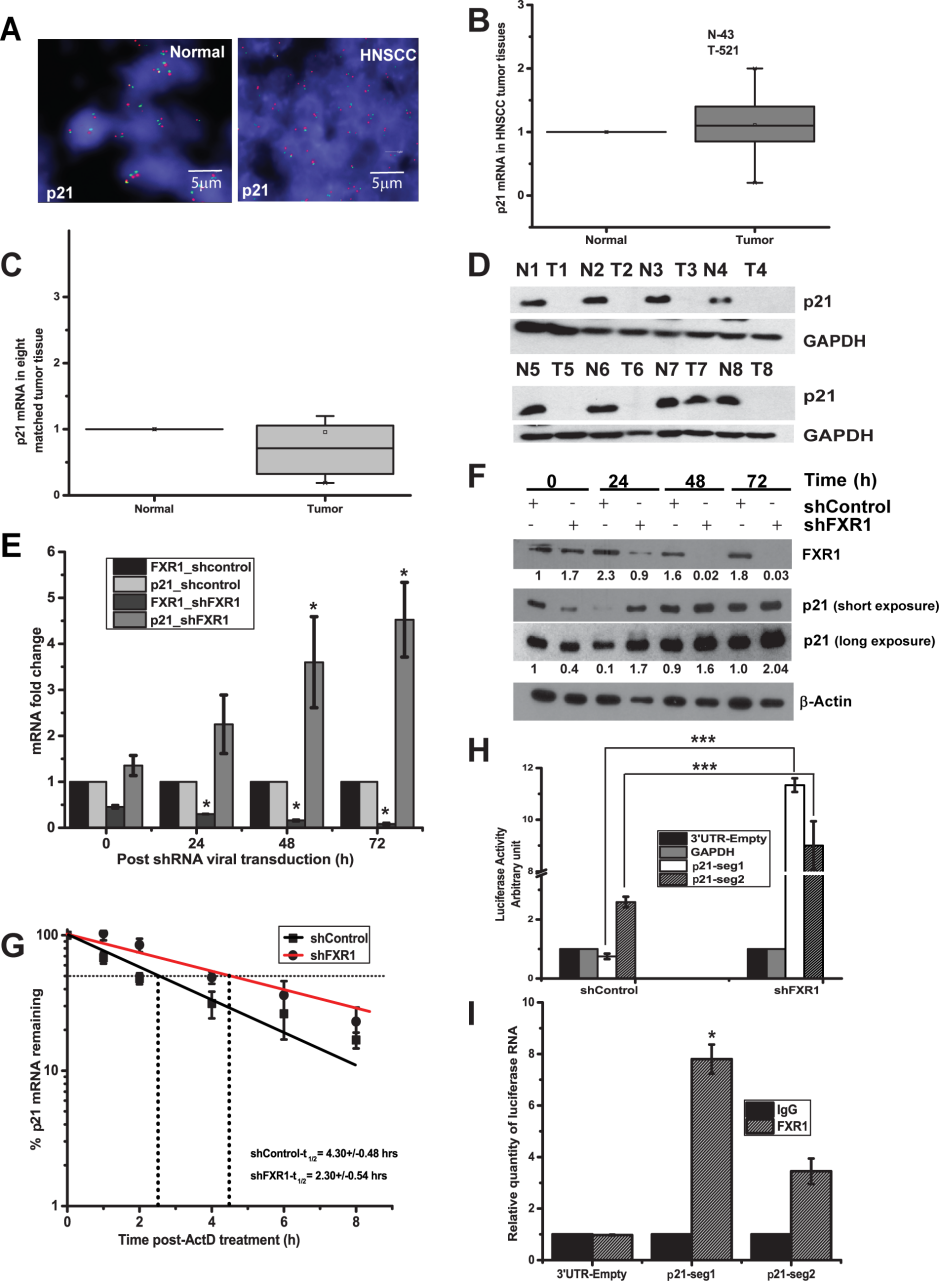
FXR1 destabilizes *p21* mRNA. (A) FISH analysis of p21 in a HNSCC TMA. Green indicates p21 and red denotes the control loci (scale bar 5μm). (B) Relative *p21* expression data obtained from cancer genome browser (N-43, T-521) (S4 Table). (C) Relative mRNA quantity of *p21* in eight matched HNSCC tumor compared to normal adjacent tissue samples estimated using qRT-PCR. GAPDH serves as a control. (D) Immunoblot analysis of p21 protein from eight representative matched HNSCC tumor and normal adjacent samples. GAPDH is used as a loading control. (E) Relative quantity of *FXR1* and *p21* levels estimated by qRT-PCR in UMSCC74B cells after treatment with FXR1 shRNA. Cells were collected at indicated time points. *FXR1* and *p21* levels in shControl treated UMSCC74B cells were taken as 1 for each time points. (F) Immunoblot analysis of p21 protein at different time points, as, mentioned in Fig 4E. (G) The mRNA decay rate of *p21* as indicated in UMSCC74B cells by qRT-PCR after silencing FXR1 followed by transcription inhibition with actinomycin-D for mentioned time points in the graph. Actin serves as a control. Data here represents the mean of n  =  3 experiments. (H) Forty-eight hours after transfection of UM74B FXR1 KD and control cells with empty 3’UTR luciferase plasmid, luciferase-fused *GAPDH* 3′UTR plasmid or different segments of *P21* 3′UTR, the lysates were analyzed for luciferase activity using luminometer. The empty 3’UTR luciferase plasmid and luciferase-fused *GAPDH* 3′UTR served as a transfection and loading control. Values are the means ± SD from three independent experiments by using unpaired two sample t-test. (I) Binding of FXR1 with the 3′UTR of *p21seg1* and *p21seg2* RNAs at the G4 region. RNP IP was performed 48 h post-transfection of UMSCC74B cells with *seg1* and *seg2* 3′UTR fused to a luciferase reporter construct. Luciferase mRNA was detected using qRT-PCR. The luciferase gene in the empty-3′UTR was used as a transfection and qRT-PCR control. (*p<0.05, **p<0.005, ***p<0.0005).

First, to estimate the levels of p21 at the DNA copy number, we used FISH analysis in HNSCC TMA. As shown in Fig 4A, compared to normal (left panel), p21 is not amplified in HNSCC TMA tested in this study (S3 Table). Next, the copy number changes of p21 was verified at the mRNA level with the data obtained from Cancer Genome Browser, and as shown in Fig 4B *p21* levels are highly comparable with normal tissue samples. The expression of this mRNA is not significant in cancer genome browser. This observation is well correlated with the TCGA data, where p21 (DNA copy number and mRNA combined) expression is only 3% from 279 patients. As shown in Fig 4C, *p21* levels are downregulated, though not significantly, in eight representatives matched normal adjacent and HNSCC tumor tissue samples. Furthermore, the levels of *p21* protein were tested from the same eight representative matched HNSCC tumor and normal adjacent samples. As shown in Fig 4D, *p21* is mainly expressed in the eight normal adjacent samples compared to tumor tissues. We did observe a discrepancy in p21 mRNA and protein expression patterns in normal and tumor tissues. In addition to mRNA changes, there is a possibility that p21 protein is altered at the post-translational level. And p21 protein analyses of the tumor samples correlated with the mRNA data shown in Fig 4C. Thus, p21 is downregulated in HNSCC compared to normal adjacent tissues tested here.

Second, based on our data in Fig 3G that FXR1 binds to *p21* as well as previous report showing association of FXR1 with G4 RNA structure in the 3'–UTR region *p21* [26], we therefore probed the relationship between FXR1 and *p21* in HNSCC. Based on our RNA IP data (Fig 3G), *p21* is associated with FXR1. Hence, we planned to determine whether silencing of FXR1 influences the expression of p21 mRNA. As shown in Fig 3A, FXR1 KD has induced the expression of *p21* in both the oral cancer cell lines. Next, to test if FXR1 KD was directly correlated with *p21* mRNA expression, we used a time course assay. As shown in Fig 4E, 0hrs of post transduction with shFXR1 exhibits a steady and significant (*p<*0.05) increase in *p21* mRNA as tested until 72 hrs in UMSCC74B cells. Next, to test the changes in *p21* exerted by FXR1 in translation, we tested p21 protein levels at different time points. FXR1 KD promotes p21 protein expression over the course of 0 to 72hrs (Fig 4F). Altogether, these data indicate that silencing of FXR1 promotes the expression of p21 in oral cancer cells. To estimate the post-transcriptional changes caused by FXR1 to control cellular *p21* stability, we treated shcontrol- or shFXR1-transduced UMSCC74B cells with the transcription inhibitor actinomycin D (ActD), and measured the half-life of *p21* by qRT-PCR. Linear regression on semi-log values of *p21* mRNA decay rate in shcontrol-transfected cells provided an estimated half-life of approximately 2:30±0:54 hrs compared to shFXR1 exhibited a statistically significant (*p<*0.05, n = 3) increased half-life of 4:30±0:48 hrs (Fig 4G). Next, in order to determine the specific G4 sequences in human *p21* and their association with FXR1, first, we measured the G-scores within the RNAs. Based on high and low G-scores, we cloned G4 regions of *p21* 3’UTR in the 3’UTR of Renilla luciferase plasmid (S2C and S2E Fig). The segment 1 (seg1) of p21 3’UTR has highest G-score compared to seg2. 3’UTR of human *p21* shows different G4 containing regions with high and low G-scores compared to mouse *p21* 3’UTR where the G4 region is enriched in one location [26]. As shown in Fig 4H, the luciferase activity of seg1 in control cells is lower than seg2 because FXR1 binds and destabilizes seg1 compared to seg2. However, in the absence of FXR1 seg1 exhibits increased luciferase activity compared to seg2 which corroborates with total mRNA levels as shown in Fig 3A. Furthermore, seg2 in control cells exhibits increased luciferase activity indicating that FXR1 preferentially binds to seg1 compared to seg2 and promote destabilization of the RNA. Next, we wanted to show a direct binding of FXR1 to these luciferase constructs containing seg1 and seg2 of *p21* 3’UTR by RNP-IP assay. UMSCC74B cells are transfected independently with 3’UTR-empty or 3’UTR-p21seg1 or 3’UTR-p21seg2. 48hrs of post-transfection, cells are collected for RNP-IP analyses. As shown in Fig 4I, qRT-PCR analysis for luciferase RNA shows that seg1 binds to FXR1 more efficiently (*p<*0.05, n = 2) compared to seg2 with a low G4 sequences. As shown in S2D Fig, qRT-PCR for luciferase in these input samples shows that the transfection efficiency is comparable in all samples.

Thus, silencing of FXR1 in oral cancer cells facilitates an increase in steady-state level of *p21* and subsequently promotes its protein expression, indicates that overexpressed FXR1 in HNSCC destabilizes *p21* mRNA and reduces its protein expression.

### FXR1 stabilizes *TERC* RNA

**Fig 5:**
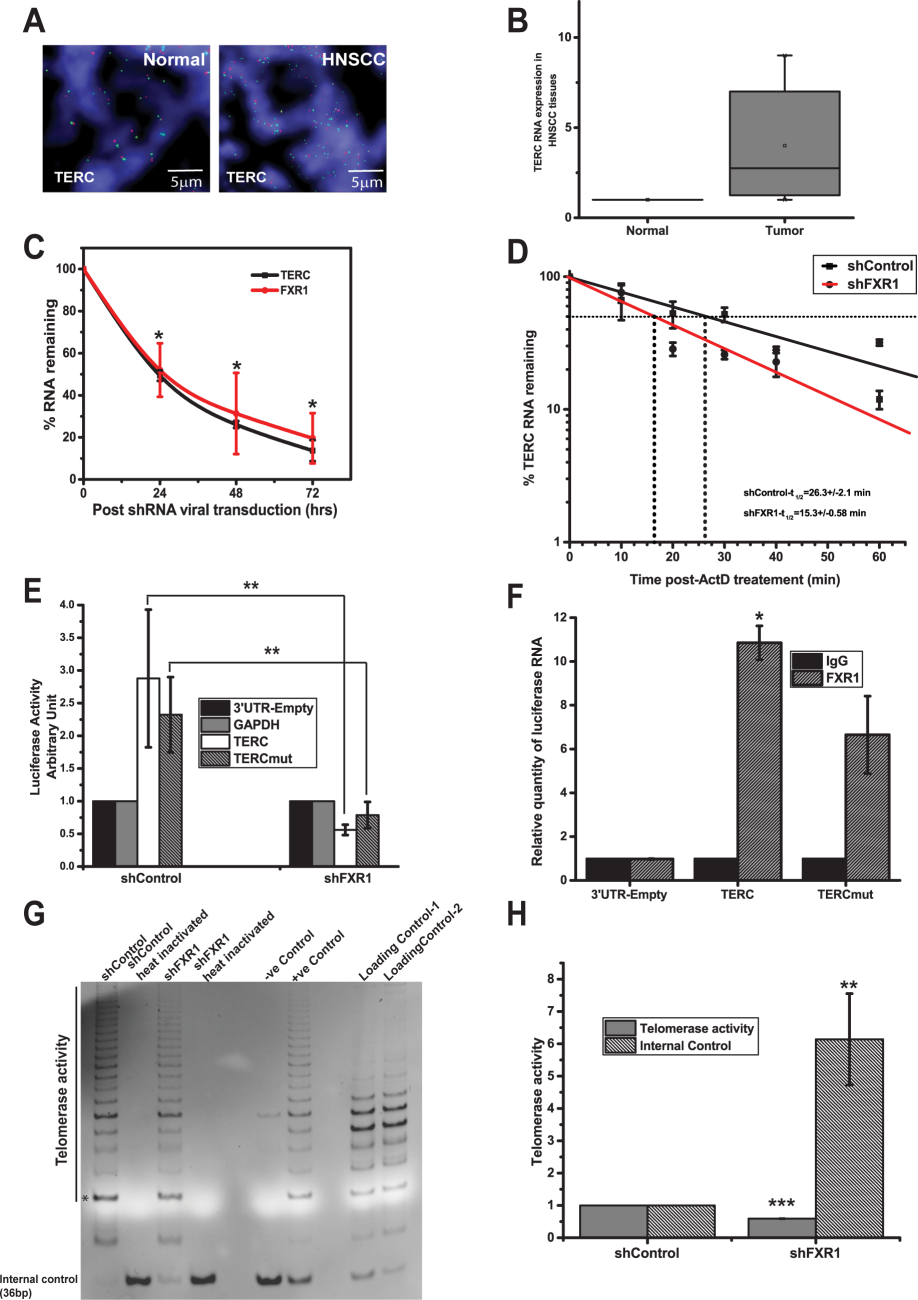
FXR1 stabilizes *TERC* RNA. (A) FISH analysis of *TERC* DNA in a HNSCC TMA. Green indicates *TERC* DNA and red denotes control loci (scale bar 5μm). (B) Relative expression of *TERC* estimated by qRT-PCR in eight matched HNSCC and normal adjacent tissue samples. (C) Relative expression of *FXR1* and *TERC* levels are simultaneously estimated in UMSCC74B cells under treatment of FXR1 shRNA for indicated time points. *FXR1* and *TERC* levels in shControl treated UMSCC74B cells were taken as 100% for each time points. (D) The RNA decay rate of TERC as indicated in UMSCC74B cells by qRT-PCR after silencing FXR1 followed by transcription inhibition with actinomycin-D for mentioned time points in the graph. Actin serves as a control. Data here represents the mean of n  =  3 experiments. (E) Forty-eight hours after transfection of UM74B FXR1 KD and control cells with empty 3’UTR luciferase plasmid, luciferase-fused *GAPDH* 3′UTR plasmid or two segments of *TERC*, the lysates were analyzed for luciferase activity using luminometer. The empty 3’UTR luciferase plasmid and luciferase-fused *GAPDH* 3′UTR served as a transfection and loading control. Values are the means ± SD from three independent experiments by using unpaired two sample t-test. (F) Association of FXR1 with the *TERC* and *TERCmut* RNAs. RNP IP was performed 48 h post-transfection of UMSCC74B cells with *TERC* and *TERCmut* fused to a luciferase reporter construct. Luciferase mRNA was detected using qRT-PCR. The luciferase gene in the empty-3′UTR was used as a transfection and qRT-PCR control. (G) TRAP assay is done on protein extracts (0.05μg) from FXR1 KD and control UMSCC74B cells. Heat inactivated controls; negative, positive, and loading controls are also shown in the figure. (H) Quantification of internal controls and telomerase activity bands (*) from FXR1 KD and control UMSCC74B cells. The values were normalized against the internal control bands from the heat inactivated samples from the above mentioned samples. *P* values are calculated from 4 independent experiments. (*p<0.05, **p<0.005, ***p<0.0005).

First, we determined the TERC DNA copy number (chromosomal locus is 3q26.2) status which is independently verified by FISH analysis in a HNSCC TMA. As shown in Fig 5A, compared to normal (left panel), TERC DNA is amplified in multiple loci of the tumor tissue samples (Fig 5A and S3 Table). Next, we tested the RNA levels of *TERC* in eight tumor tissue samples compared to normal adjacent tissues. Our analyses indicate that *TERC* is overexpressed in HNSCC tumor tissues compared to normal adjacent tissues (Fig 5B). A similar trend is also observed in TCGA where the combined DNA copy number and RNA expression is amplified in 25% of 279 tissue samples. Collectively, these observations indicate that *TERC* is overexpressed in HNSCC tissues.

Second, our initial analysis presented above demonstrates that FXR1 depletion correlates with downregulation of *TERC*. Given that *TERC* associates with FXR1, we sought out to determine whether FXR1 directly regulates *TERC* accumulation in HNSCC cells by utilizing RNA turnover pathway. To understand the correlation between *FXR1* and *TERC* levels over the course of the time, we tested both *FXR1* and *TERC* simultaneously in FXR1 KD UMSCC74B cells. As shown in Fig 5C, an equally steady decrease of both *TERC* and *FXR1* is observed under FXR1 depleted oral cancer cells. To estimate the post-transcriptional changes caused by FXR1 to control endogenous *TERC* stability, we treated shcontrol- or shFXR1-transduced UMSCC74B cells with the transcription inhibitor actinomycin D (ActD), and measured the half-life of *TERC* by qRT-PCR. Linear regression on semi-log values of *TERC* mRNA decay rate in shFXR1-transfected cells provided an estimated half-life of approximately 15.3±0:58 min compared to shControl exhibited a statistically significant (*p<*0.05, n = 3) half-life of 26.3±2.1 min (Fig 5D). Taken together, these observations possibly suggest that the observed upregulation of *TERC* in HNSCC primarily at the post-transcriptional level through increased RNA stability that is mediated by FXR1. Next, in order to determine the specific G4 sequences and their association with FXR1, first, we measured the G-scores within the TERC RNA. Based on high and low G-scores, we cloned full-length and mutant *TERC* at 3’UTR of Renilla luciferase plasmid (S2C and S2E Fig). When full-length and truncated *TERC* (28 base deletion at 5’-end, TERCmut) (S2C and S2E Fig) are used for luciferase assay, TERC exhibits increased luciferase activity in control cells compared to TERCmut (Fig 5E). However, in FXR1 KD cells, TERC exhibits a decreased luciferase activity compared to TERCmut indicating that FXR1 binds to specific G-rich sequences in these RNAs and possibly controls their turnover. Next, we wanted to establish a direct binding of FXR1 to these luciferase constructs containing TERC and TERCmut by RNP-IP assay. UMSCC74B cells are transfected independently with 3’UTR-empty or 3’UTR- TERC or 3’UTR-TERCmut. 48hrs of post-transfection, cells are collected for RNP-IP analyses. As shown in Fig 5F, qRT-PCR analysis for luciferase RNA shows that full-length TERC binds to FXR1 more efficiently (*p<*0.05, n = 2) compared to TERCmut with low G4 sequences. As shown in S2D Fig, qRT-PCR for luciferase in these input samples shows that the transfection efficiency is comparable in all samples. As *TERC* deregulation is often associated with telomere length [38], down regulation of *TERC* in HNSCC cells prompted us to determine the telomerase activity by using TRAPeze® Telomerase Detection Kit (Millipore, USA). FXR1 KD cells showed an appearance of the internal control (36bp) band compared to control which is correlated with a reduced telomerase activity (Fig 5G and 5H). We quantified both the internal control (36bp) and the first telomerase activity (*) bands in shControl and shFXR1 lanes. The data was normalized against the internal control bands from the two heat inactivated sample lanes for each experiment set, respectively (Fig 5F). Thus, silencing of FXR1 reduces the level of *TERC* and subsequently interferes with the telomerase activity. Altogether, these data indicate that overexpression of FXR1 in HNSCC play a key role in stabilizing *TERC* to bypass cellular senescence.

### FXR1 evades senescence by a pathway involving both p21 protein and *TERC*

**Fig 6:**
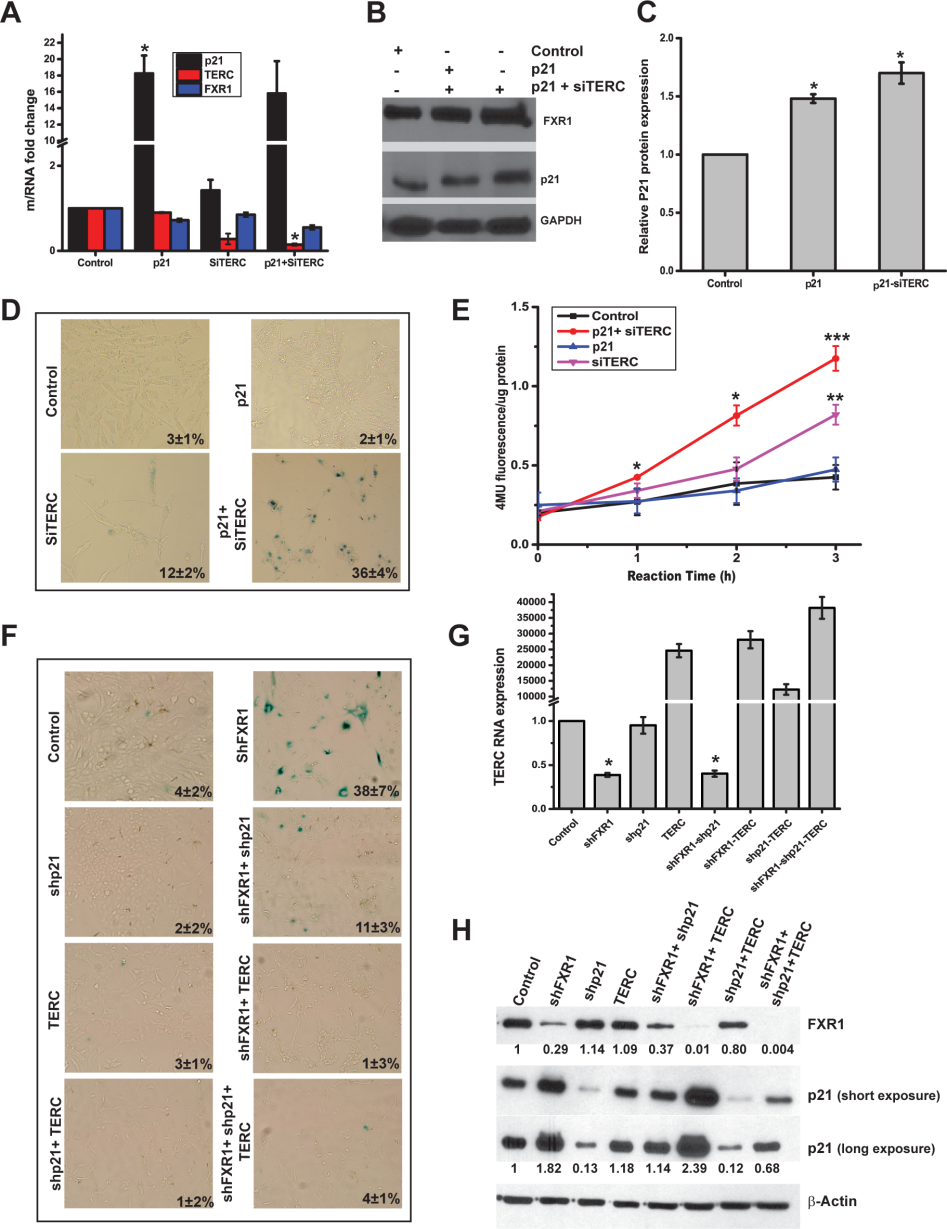
FXR1 involves both p21 and *TERC* RNA to promote senescence. (A) qRT-PCR analyses of RNA levels of UMSCC74B cells transfected with single or combination of p21 overexpression plasmid or si*TERC* RNA. GAPDH serves as a loading control. (B) Immunoblot of protein expression levels of FXR1 and p21 in UMSCC74B cells transfected independently or together with p21 overexpression plasmid or si*TERC*. GAPDH serves as a loading control. (C) Quantification of the western blot shown in Fig 6B. (D) Staining of SA-β-gal in UMSCC74B cells transfected independently or together with p21 overexpression plasmid or si*TERC*. (E) Quantitative values of *MUG* conversion to 4-MU by senescence associated β-galactosidase for Fig 6D. (F) SA-β-gal staining of shControl and shFXR1 treated UMSCC74B cells, also co-transduced and/or co-transfected together or independently with shp21 or TERC overexpression plasmid. (G) qRT-PCR analyses of TERC RNA levels in shControl and shFXR1 treated UMSCC74B cells also co-transduced and/or co-transfected together or independently with shp21 or TERC overexpression plasmid. GAPDH serves as a loading control (n = 2). Statistical significance for TERC overexpressing cells from a plasmid-borne copy is not calculated. (H) Immunoblot of protein expression levels of FXR1 and p21 in shControl and shFXR1 treated UMSCC74B cells also co-transduced and/or co-transfected together or independently with shp21 or TERC overexpression plasmid. β-Actin serves as a loading control. (*p<0.05, **p<0.005, ***p<0.0005).

Based on the two independent experiments described above, FXR1 concurrently destabilizes *p21* (Fig 4E–4G) and stabilizes *TERC* (Fig 5C and 5D) to repress cellular senescence in HNSCC. Upregulation of p21 induces cell cycle arrest during replicative senescence in cell culture [39,40]. In addition, *TERC* is an essential RNA component of the telomerase enzyme complex that has been directly implicated in the maintenance of telomere length and in the prevention of premature senescence and aging. In support of this function, *TERC*-deficient mice displayed short telomeres, chromosomal instability, and premature aging [41]. To test whether these two coordinated events such as down-regulation of p21 and up-regulation of *TERC* in cancer cells, independently or in combination to control senescence, we overexpressed p21 and silenced *TERC* by individual and combinatorial transfections in HNSCC cells. Overexpression of p21 by plasmid transfection and silencing of *TERC* by siRNA were verified by qRT-PCR analyses. As shown in Fig 6A, *p21* is overexpressed more than 18-fold (*p<*0.05) and *TERC* is significantly (*p<*0.05) downregulated in oral cancer cells. We see changes in *FXR1* levels after these transfections but the protein levels did not change. In addition, silencing *TERC* by siRNA does not alter the expression of p21 (Fig 6A). We further confirmed the levels of FXR1 and p21 protein levels in UMSCC74B cells (Fig 6B, additional cell line UMSCC74A- S3A and S3B Fig); and the protein expression is also quantified as a mean of two separate western blots (Fig 6C). The data indicate that ectopic expression of *p21* increases the expression of p21 protein significantly without altering the levels of FXR1 or *TERC*. Next, to study whether these changes alter cellular senescence, we stained the cells with SA-β-gal. The data indicate that, independent overexpression of p21 did not show SA-β-gal staining, however, silencing of *TERC* showed weak staining (Fig 6D) (additional cell line UMSCC74A- S3C Fig). Quantitation of *MUG* conversion to 4-MU by senescence associated β-galactosidase for Fig 6D is shown in Fig 6E. To further confirm that FXR1 is a key regulator of senescence in these oral cancer cell lines and it does so by modulation p21 and TERC RNA, we set up the following experiment. As shown in Fig 6F, we treated UMSCC74B cells with shControl and shFXR1. And at the same time, the treated cells are also treated with shp21 and/or transfected with TERC overexpression plasmid. 72h of post-treatment, the treated cells were stained with SA-β-gal. Strong SA-β-gal staining was observed again in shFXR1 only treated cells. Light staining was also observed in cells treated with both shFXR1and shp21. Here, the light staining with SA-β-gal is similar to our observation in Fig 6D where the use of siTERC showed light staining in UMSCC74B cells. TERC RNA expression was estimated in all the plasmid transfected and/or shRNA transduced cells (Fig 6G). As shown in Fig 6H, FXR1 and p21 protein levels were determined in the treated cells by western blot analyses. Nevertheless, concurrent overexpression of p21 and silencing of *TERC* significantly promoted senescence evidenced by staining of SA-β-gal (Fig 6D). Furthermore, the 4MU fluorescence per microgram are significantly high (UMSCC74A, *p<*0.05 and UMSCC74B, *p<*0.005) in those with concurrent overexpression of p21 and silencing of *TERC* compared to independent changes (Fig 6E and S3D Fig). Taken together, these data indicate that FXR1-regulated repression of senescence involves both down-regulation of p21 and up-regulation of *TERC* in oral cancer cells.

### FXR1-mediated senescence is irreversible

**Fig 7:**
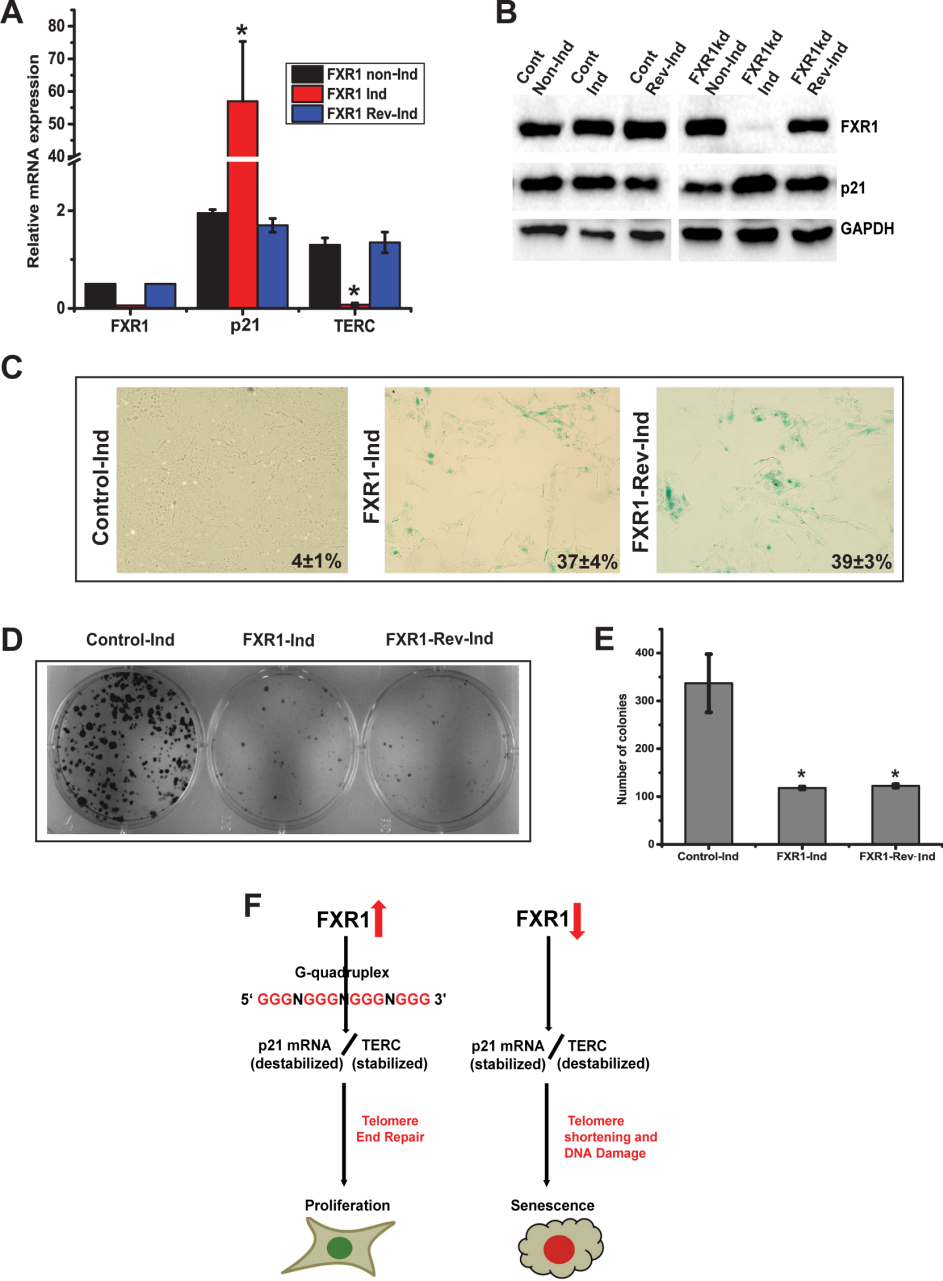
FXR1-regulated senescence is irreversible. (A) Relative quantity of *FXR1*, *p21*, and *TERC* are quantified by qRT-PCR under non-inducible, Inducible and reverse inducible FXR1 silencing conditions in UMSCC74B cells. (B) Immunoblot analysis of protein levels of UMSCC74B cells as described above. (C) The UMSCC74B cells are subjected to SA-β-gal staining as described in Fig 7A. (D) Representative culture dishes from clonogenic assays of cells transfected with indicated conditions. (E) The panel depicts the colony forming efficiency from clonogenic assays of UMSCC74B cells. The data are presented as the means ± S.D. from three independent experiments. (F) Model representation of evasion of cellular senescence through FXR1 by destabilizing p21 and stabilizing *TERC* in conjunction with activation of p53. (*p<0.05, **p<0.005, ***p<0.0005).

Senescence has been shown to be either reversible or irreversible depending on cell type and exposure to cytotoxic agents [42,43]. To test whether FXR1-dependent senescence is reversible or irreversible, we used inducible clones of shcontrol and shFXR1 to silence FXR1 under Isopropyl β-D-1-thiogalactopyranoside (IPTG, 0.5mM) promoter. First, the stable UMSCC74B cells, transfected with shcontrol and shFXR1 (MISSION 3X LacO Inducible Non/Target shRNA), were treated with 1mM IPTG for 6 days to test the phenotype. Second, IPTG was removed from the medium to induce FXR1 expression back in the cells. The cells were then grown for additional 6 days to test senescence. Under both the conditions, the levels of FXR1, p21, and *TERC* are quantified by qRT-PCR. As shown in Fig 7A, IPTG-shFXR1 treated cells indicate that both FXR1 mRNA and *TERC* are significantly (*p<*0.05) downregulated and p21 mRNA is significantly (*p<*0.05) upregulated compared to non-induced control cells. However, when IPTG is removed, the RNA levels return back to comparable levels with the non-induced control cells (Fig 7A). Next, the protein levels were measured to ensure that the FXR1 shRNA inducible cells respond to the treatment of IPTG. Protein p21 is increased as FXR1 protein is silenced under the treatment of IPTG compared to control and IPTG removed cells (Fig 7B). Next, we have stained the cells with SA-β-gal to estimate the senescence. The SA-β-gal staining shows an appearance of bluish green stain in both IPTG incubated and removed FXR1 KD cells compared with induced control (Fig 7C). To note, the IPTG removed cells express FXR1 protein comparable to induced control cells (Fig 7B), hence, it was interesting to test whether the cells are reverting back from senescence by estimating their proliferation ability and survival through colony formation assays. As expected, the IPTG incubated FXR1 KD cells fail to form colonies after 18 days (longer time is given to form adequate number of colonies). Surprisingly, IPTG removed FXR1 expressing cells also fail to form colonies in comparison with non-induced control cells which form large colonies (Fig 7D). The number of colonies are reduced from 337 to 118 (35% decrease) upon FXR1 KD. Moreover, IPTG removed FXR1 expressing cells also exhibit reduced colony number of 123 (36% decrease) in UMSCC74B cells (Fig 7E). Taken together, these data indicate that FXR1-regulated senescence is not reversible in oral cancer cells, even after expressing FXR1 back into the cultured cells.

## Discussion

Post-transcriptional control of gene expression is gaining much attention due to the fact that RBPs are critical regulators of genes involved in DDR and genome instability [9]. Till date, there are few RBPs that have been implicated in DDR and functionally involved in promoting or suppressing cellular senescence [44]. In this report, we show that knockdown of RBP FXR1 is a major factor involved in cell cycle arrest and promoting cellular senescence through turnover of two distinct RNA targets in oral cancer cells. These findings indicate that in addition to changing the transcriptional landscape of RNAs through DDR, overexpression of certain RBPs are critically involved in increasing the life span of cancer cells. As RBPs are widely recognized for their extensive roles in several cancer biological processes such as survival, apoptosis and metastasis [45,46], a better understanding of RBPs role in cellular senescence will provide a new insight into the post-transcriptional gene regulation.

Firstly, cellular senescence, one of the hallmarks of cancer and aging, can be induced by telomere dysfunction that specific DNA damage, chromatin instability, and oncogene activation [47]. Our findings indicate, specific shRNA mediated knockdown of FXR1 induces the expression of mRNAs of p53, p27 and p21 and their encoding proteins (Fig 3A and 3B). An increased stabilization of p21, in particular, is a marker of cancer cell senescence [48]. An increased p21 protein levels is also associated with reduced cell growth in cancer [49]. Interestingly, it has been noted that in mouse C2C12 myoblasts a reduced level of FXR1 promotes p21 expression by association with G4-RNA structures present in the 3' UTR of *p21* [26]. Taken together it suggests that the overexpression of FXR1 protein in cancer may aid in an important mechanism for evasion of cellular senescence through reduced mRNA and protein levels of p21. Although recent work that examined FXR1 in human cancers showed silencing of FXR1 exhibited reduced cancer cell growth in vitro and in vivo [23], the precise molecular mechanism of FXR1-regulated cancer cell growth was not addressed. Our proposed model (Fig 7F) demonstrates that overexpression of FXR1 post-transcriptionally facilitates p21 mRNA destabilization and reduces its expression in HNSCC, possibly promoting cancer cell proliferation.

Secondly, cancer cell senescence is shown to be associated with DDR and correlated with p53-mediated gene expression patterns and also telomere shortening in multiple cancer models [44,50,51,52]. Here, we show that, loss of FXR1 results in DNA damage (Figs 2D, 2E and 3F), induces p53 mRNA and protein (Fig 3A and 3B), and ultimately resulting in senescence in oral cancer cells. Increase in p53 and p21 are correlated with DDR-induced senescence [3,31], and our data demonstrate that silencing of FXR1 promotes double stranded DNA breaks by yH2AX foci formation (Figs 2D, 2E and 3F). In addition, upregulation of p21 by the p53 tumor suppressor gene has been well documented [39,49,53]. Here, we show that FXR1-mediated downregulation of p21 plays a role in repressing p53-dependent cellular senescence. Furthermore, our data indicates that p53 appears to play a critical role in p21 induction in FXR1 depleted cells (Fig 3C–3E). In addition, we have observed destabilization of *TERC* and slightly reduced telomerase activity (Fig 5C–5I) which indicates that *TERC* degradation could be an important biological process that promotes senescence in part with p21. Treatment with ActD shows a significant decrease in TERC RNA half-life in FXR1 KD cells (Fig 5D). This data refers that FXR1 plays a role in the stability of *TERC* which is consistent with previous work identifying RBP Dyskerin as a key regulator of *TERC* that in turn controls the mammalian telomerase activity [54,55,56]. Interestingly, Dyskerin is the only well-characterized RBP that has shown to bind to *TERC*, but our study has now identified FXR1 as another controller of *TERC* which binds and stabilizes the RNA. Additional work is needed to warrant the recruitment of *TERC* by FXR1 and how this process controls telomere length in cancer.

Finally, studies demonstrate a link between p21 and *TERC*, increased telomere erosion, and DDR. For example, knockout of *TERC* causes progressive telomere shortening that persistently activates DDR and leads to numerous abnormalities in stem cell function and accelerated aging [57,58]. At the cellular level, DDR promotes a permanent cell-cycle arrest and initiates senescence [31,59]. Interestingly, loss of p21 in *TERC* -null mice with dysfunctional telomeres leads to improved stem cell function and increased lifespan without accelerating tumor formation [60]. In this regard, our findings provide a physiological basis by which FXR1 prevents cellular senescence through loss of p21 and upregulation of *TERC*. Our data also indicate that, overexpression of p21 alone is not sufficient for promoting senescence in oral cancer cells (Fig 6D and 6E); it also requires down-regulation of *TERC* (Fig 6D and 6E). This is also clear from Fig 6F where senescence is observed when only FXR1 alone is knocked down. Correlative with Fig 6D and 6E, we also see some senescent cells where both FXR1 and p21 are knockdown suggesting down-regulation of TERC can still bring about senescence but at a lower degree. RBPs play a major role in genome instability and DDR to control gene expression patterns [45,61,62]. The action of DDR-mediated activation of p53/p21 activated senescence requires down-regulation of *TERC* by FXR1 which also provides a basis for RBPs’ role in DDR and genome instability. Studies strongly support that G-4 RNA structures present in telomere RNAs [63,64], play a critical role in promoter activity as well as its expression. Our observation on G4 RNA structures, which are strongly enriched in 3' UTR sequences including that of p21 and full length *TERC* (more the G-score stronger the G4 structure, Figs 4H and 4I, 5F and 5G and S2E Fig), provides an opportunity to shed light on their importance in senescence and aging. Our data clearly implicate that both G4 structure containing *p21* and *TERC* bind to FXR1 (Figs 4H and 4I, 5F and 5G and S2E Fig), providing a basis for post-transcriptional control of two distinct mechanisms (Fig 7F). Cellular senescence is considered irreversible in the sense that known physiological stimuli cannot force senescent cells to re-enter the cell cycle [59]. Our data supports that FXR1-regulated elucidation of senescence is irreversible based on the colony formation assay (Fig 7D and 7E). Furthermore, FXR1 promotes cellular senescence in WT p53 expressing cells compare to p53 mutant stable HNSCC cells (Fig 3C–3E). Thus, FXR1 possibly plays a role in suppressing p53 for checkpoint control. Therefore, our data highlight an important unifying role of FXR1 towards the p53/p21-*TERC*-pathway in dictating cell growth over cellular senescence in HNSCC (Fig 7F).

## Materials and Methods

### Biospecimens

Human tissues were obtained from Hollings Cancer Center (HCC) biorepository with written informed consent and local MUSC Internal Review Board approval (Pro00009235 (CT)#101547). Frozen tumor tissues are micro-dissected to assure that > 80% of tumor contained HNSCC. The HNSCC and normal adjacent tissues contained tissue microarrays that are used for the evaluation of FXR1 and p21, and TERC DNA expression using FISH. Samples are subjected to protein and RNA extraction for immunoblotting and qPCR analyses, respectively.

### Cell lines, reagents and antibodies

HNSCC cell lines UMSCC-11A, -11B, -74A and -74B were obtained from University of Michigan and SCC4, SCC9, SCC25 and CAL27 were obtained from ATCC. Cell lines were routinely grown in Dulbecco’s modified Eagle medium (DMEM-Hyclone) containing 10% fetal bovine serum (FBS) with 100U/ml penicillin and 100 μg/ml streptomycin. HOK cells (Science Cell) were grown in keratinocyte serum-free medium supplemented with BPE and EGF (Gibco, BRL). SCC cell lines were grown in DMEM: F12 (1:1) containing 400 ng/ml hydrocortisone, 10% FBS, and 100U/ml penicillin and 100 μg/ml streptomycin. Different shRNA constructs for FXR1 (TRCN0000158932 and TRCN0000159153) and p21 (TRCN0000287021) were obtained from Sigma Mission. FXR1 inducible shRNA clone with TRCN0000159153 was obtained from Sigma Mission. p21, pCEP-WAF1 was a gift from Bert Vogelstein (Addgene plasmid # 16450) and TERC, pBABEpuro U3-hTR-500 was a gift from Kathleen Collins (Addgene plasmid # 27666), over-expression plasmids are obtained from Addgene. siTERC RNAs (100uM; GGGCGUAGGCGCCGUGCUU and CCCACUGCCACCGCGAAGA) were purchased from Sigma Mission whereas control siRNA (20nM; GTTCAATTGTCTACAGCTA) was from Dharmacon RNAi Technologies. Regular transfection was done with lipofectamine-2000 (life technologies). siRNA transfections were done with HiPerfect (QIAGEN) transfection reagent, following the manufacturer’s protocol. FXR1 antibodies were obtained from Cell Signaling Technology (CST) and EMD Millipore, FMR1 was from Abgent and FXR2 was from Bethyl Laboratories. p21 and p27 antibodies were from BD Pharminogen. P53 was from Santa Cruz. AKT, p-AKT (S473), PTEN, γ-H2AX, and GAPDH were from CST. β-Actin was purchased from Sigma. Alexa Fluor 488 was bought from life technologies. SA-β-gal assay kit was purchased from CST. MUG was purchased from Sigma-Aldrich. Horseradish peroxidase-conjugated anti-mouse and anti-rabbit immunoglobulinG were procured from GE Healthcare Biosciences (Uppsala, Sweden). Protein A/G beads were purchased from Santa Cruz Biotechnology. Fugene HD transfection reagent and LightSwitch Luciferase Assay kit (LS010) were purchased from Switchgear Genomics.

### Fluorescence in Situ Hybridization (FISH)

FISH assays are performed on unstained TMA sections. BAC clones *(FXR1*: *BAC clones*: *RP11-314I4 (green) RP11-480B15 (red); p21*: *RP11-265F6 (green) RP11-624F22 (red); TERC RNA*: *RP11-990E14 (green) RP11-480B15 (red)* are selected from the UCSC genome browser and purchased through BACPAC resources (Children's Hospital, Oakland, CA). Following colony purification DNA is prepared using QiagenTips-100 (Qiagen, Valencia, CA). DNA is labeled by nick translation method with biotin-16-dUTP and digoxigenin-11-dUTP for 3' and 5' probes and locus and control probes respectively (Roche, USA). Probe DNA is precipitated and dissolved in hybridization mixture containing 50% formamide, 2X SSC, 10% dextran sulphate, and 1% Denhardt's solution. Approximately 200ng of labeled probe is hybridized to normal human chromosomes to confirm the map position of each BAC clone. FISH signals are obtained using anti-digoxigenin-fluorescein and AlexaFluor-594 conjugate to obtain green and red colors, respectively. Fluorescence images are captured using a high resolution CCD camera controlled by ISIS image processing software (Metasystems, Germany).

### Qualitative in situ and quantitative SA-β-gal assay

SA-β-gal activity is measured according to the manufacturer’s instructions (Cell Signaling Technology, Beverly, MA, USA). SA-β-Gal activity is detected using X-gal (5-bromo-4-chloro-3-indolyl β-D-galactoside) staining at pH 6.0 at 72 hours post-transduction with shRNAs unless otherwise mentioned. Using light microscope, three representative fields are captured under white light for three independent experiments. The senescence associated β-gal activity in UMSCC74A and UMSCC74B is quantified by a method as described elsewhere [32]. Briefly, SA-β-gal is measured by the rate of conversion of 4-methylumbelliferyl-α-D-galactopyranoside (MUG) to a fluorescent hydrolysis product 4-methylumbelliferone (4-MU) at pH 6.0. Treated UMSCC74A and UMSCC74B cells grown in 60-mm plates are washed three times with Hank’s balanced salt solution. Cells are then lysed by 200μl of lysis buffer, scraped, transferred to a 1.5-ml tube, vortexed, and centrifuged at 12,000*g* for 5min. The clear supernatant is then used for the assay after measuring the total protein by Biorad spectrophotometer. Reaction buffer at 2X strength is mixed with 1.7mM of MUG added immediately prior to use from a 34mM stock in dimethyl sulfoxide. For final reaction the 2X reaction buffer (150μl) is mixed with 150μl of clarified lysate (100μl of lysate diluted with 50μl of lysis solution) and carried out at 37°C water bath for 0, 1, 2, and 3 hours. At the end of each time points the reaction is stopped with 400mM sodium carbonate. The stopped reaction mixture is read by using150μl per well in a 96-well plate using a plate reader with excitation at 385 nm, emission at 465nm, and gain held constant at 460. Normalized SA-β-gal activity is expressed as observed fluorescence divided by micrograms of total protein in the assay.

### FXR1 RNP IP (RIP) and qRT-PCR

FXR1 RNP IP is performed as previously described [36] with some modifications. Briefly, cell lysates are prepared from exponentially growing UMSCC74B cells. Equal amounts of protein are used (750–1000μg). FXR1 monoclonal antibody (Millipore) or isotype control IgG (Santa Cruz) are pre-coated onto protein A/G Sepharose beads (PAS) and extensively washed using NT2 buffer [50mM Tris–HCl, 150mM NaCl, 1mM MgCl_2_, 0.05% Nonidet P-40 (NP-40), pH 7.4]. Individual pull-down assays are performed at 4°C for 1–2 h to minimize potential reabsorbing of mRNAs. For RNA analysis, the beads are incubated with 1ml NT2 buffer containing 20 U RNase-free DNase I (15 min, 30°C), washed twice with 1ml NT2 buffer and further incubated in 1 ml NT2 buffer containing 0.1% SDS and 0.5mg/ml proteinase K (15 min, 55°C) to digest the proteins bound to the beads. RNA is extracted using phenol and chloroform, and precipitated in the presence of glycogen. For analysis of individual mRNAs, the RNA isolated from the IP is subjected to reverse transcription (RT) using random hexamers and SuperScriptII reverse transcriptase (Biorad).

### RNA extraction and qRT-PCR

Total RNA is prepared from oral cancer tissues and HNSCC cell lines using Trizol (Ambion) or RNeasy mini kit (QIAGEN) by following manufacturer’s protocol. qRT-PCR for all m/RNA targets is performed using an Applied Biosystems StepOne Plus system with the SYBR green master mix RT-PCR kit (SA Biosciences). Primer sequences are provided in S6 Table.

### Cell cycle analysis

The cell cycle analyses of UMSCC74A and 74B-FXR1 knockdown cells was performed by flow cytometry using propidium iodide. After 72hrs of shRNA treatment, a total of 50,000 FXR1 KD and control cells were fixed and stained with propidium Iodide (PI), and analyzed by fluorescence-activated cell sorting (BD Fortessa X-20 Analytic Flow Cytometer) to evaluate the number of cells in different stages of cell cycles. Cell cycle analysis was done using the ModFit LT software.

### Immunofluorescence

UMSCC74A and 74B-FXR1 knockdown and control cells were washed and fixed with 4% paraformaldehyde. Fixed cells were blocked with 10% normal donkey serum followed by incubation with yH2AX and pATM primary antibodies for 1hr. Finally, the cells were washed and incubated with Alexa Fluor 488 secondary antibody and 4, 6-diamidino-2-phenylindole. Stained cells were subjected to analysis by using Olympus BX61 Microscope with Green and *DAPI*-*FITC filter*.

### Luciferase assays

The shRNA mediated 74B-FXR1 knockdown and control cells were used for luciferase assay. Different segments of human 3′UTRs of *p21*, *TERC*, and *GAPDH* were systematically identified (S2C and S2D Fig) based upon the G-scores (QGRS mapper tool) and cloned into a luciferase reporter vector system, pLightswitch-3’UTR from Switchgear Genomics. The segments were cloned between Nhe1 and Xho1 sites to express chimeric m/RNAs spanning two of the luciferase *p21* 3′UTR segments and TERC RNAs based upon high and low G-scores. The luciferase *GAPDH* 3′UTR and 3’UTR empty vector negative controls were included in all assays. Each construct was transfected in triplicates separately with either 74B-FXR1 knockdown and control cells with Fugene HD transfection reagent. Plates were incubated at 37°C for 48 h post-transfection before being removed. 100 μl of luciferase assay (buffer + substrate) reagent (LightSwitch Luciferase Assay) was added to each well of 96 well solid bottom white plates, and was incubated at room temperature for 30 min. Luminescence was measured by using a VICTOR^3^ 1420 Multilabel Counter (PerkinElmer) and the data obtained was normalized using Lightswitch normalization protocol using *GAPDH* 3′UTR and 3’UTR empty vector controls.

### Cell viability and colony formation

Cell viability rate upon FXR1 KD in UMSCC74A and UMSCC74B cells are determined using MTT cell proliferation assays (Invitrogen). Briefly, post-shRNA transfected 5×10^3^ cells were inoculated into each well of a 96-well plate (well area = 0.32cm^2^). Right after plating (considered as 0hr) and after that every 24h, medium was replaced with experimental medium (100μl). MTT solution was prepared fresh (5 mg/ml in H_2_O), filtered through a 0.22-μm filter, and kept for 5 min at 37°C. The MTT solution (10μl) was added to each well, and plates were incubated in the dark for 2 h at 37°C. Then reaction was stopped using MTT solution (10% SDS in 1N HCl) and further incubated overnight at 37°C. Next morning the absorbance was measured at *A*_570_ nm using a plate reader (Bio-Rad).

UMSCC74B inducible control and FXR1 KD (two wells) cells were counted and 1,000 cells were plated on a 6-well dish with 1mM IPTG for 9 days. After 9 days IPTG was removed from one well containing FXR1 KD cells and left for another 9 days. At the end the colonies were fixed and stained with crystal violet (0.5%w/v) in 20% methanol for 30min, plate was washed, and counted using a microscope.

### Statistical analysis

Data are expressed as the mean ± the standard deviation. Two-sample t-tests with equal variances are used to assess differences between means. Results with *p* values less than 0.05 is considered significant.

## Supporting Information

S1 Fig FXR1 KD reduces cell viability but do not show apoptosis. (A) Kaplan–Meier plots of overall survival of stage HNSCC patients (n = 522) stratified by FXR1 mRNA expression. The log-rank *P* values are shown. (B) Silencing of FXR1 in UMSCC74B cells. Cells were infected with lentivirus carrying short hairpin RNA (shRNA) against FXR1 in pLKO.1 hairpin vectors or with scrambled sequence containing vector used as control. Different multiplicity of infection (MOI) was used to titrate for the optimum knockdown. β-Actin was used as a loading control. (C) Staining for SA-β-gal in FXR1 depleted UMSCC74A and 74B cells with second shRNA directed to FXR1 (shFXR1_1 as shown in S1B Fig). (D) Relative quantity of p21 and TERC RNAs extracted from control and FXR1 KD cells (shFXR1_1) were estimated by using qRT-PCR. GAPDH serves as a control. (E) Immunoblot analysis of p21 protein in both FXR1 (shFXR1_1) depleted UMSCC74 and 74B cells. β-Actin was used as a loading control. (F) MTT analysis of cell viability in UMSCC74A and 74B cells transduced with control and FXR1 shRNA. Data presented as the mean ± SD of three experiments. (G) Western blots of FXR1 KD UMSCC74A and 74B cells for PARP and Caspase-3 cleavage. Apoptosis inducer for these cells, Doxorubicin was used to show relative PARP and Caspase-3 cleavage under drug induced apoptosis which was absent under FXR1 KD conditions. β-Actin was used as a loading control. (*p<0.05, **p<0.005, ***p<0.0005).
(TIF)

S2 Fig FXR1 KD cells display cellular senescence. (A) SA-β-Gal staining of multiple oral cancer cells as indicated treated with shRNA control and shRNA FXR1. (B) Wild type and FXR1 KO Mouse embryonic fibroblasts (MEF) were stained with SA-β-gal for senescence associated β-galactosidase activity. (C) Schematic of luciferase 3’UTR constructs used in this study. Based upon the G-score (S2E Fig) the regions were carefully selected from *p21* 3’UTR and TERC RNA. (D) qRT-PCR analyses of luciferase RNA in the input samples used for RNP-IP analyses for high and low G4 RNA containing constructs. Empty-3’UTR plasmid and/or GAPDH serve as transfection and loading control, respectively (n = 2). (E) Two G4 structures containing RNAs, 3’-UTR of *p21* and full-length *TERC* sequences were used for QGRS mapper software for determination of the G-score. Higher the G-score, stronger the G rich sequence that facilitates FXR1 binding.
(TIF)

S3 Fig Overexpression of p21 and KD of TERC RNA in UMSCC74A cells. (A) Western blot to determine the protein change in UMSCC74A cells transfected independently or together with p21 overexpression plasmid or siTERC. (B) Quantification of p21 protein overexpression in 74A cells after transfection. (C) Expression of SA-β-gal activity in UMSCC74A cells transfected independently or together with p21 overexpression plasmid or siTERC RNA. (D) *MUG* conversion to 4-MU by senescence associated β-galactosidase was measured in these transfected cells. (*p<0.05, **p<0.005, ***p<0.0005).
(TIF)

S1 Table RBP with DNA copy number variation and mutation. (DOCX)

S2 Table RBP with mRNA alteration. (DOCX)

S3 Table FISH data evaluation for FXR1, p21, and TERC in oral cancer TMA. (XLSX)

S4 Table Statistical analyses of FXR1, FXR2, FMR1, P21, and TERC m/RNA expression in tissue samples. (XLSX)

S5 Table Description of eight matched tumor tissue samples with normal adjacent. (XLSX)

S6 Table List of oligonucleotide sequences used in the paper. (DOCX)
