# Correction: RNA-Binding Protein FXR1 Regulates p21 and TERC RNA to Bypass p53-Mediated Cellular Senescence in OSCC

**DOI:** 10.1371/journal.pgen.1006411

**Published:** 2016-10-26

**Authors:** Mrinmoyee Majumder, Reniqua House, Nallasivam Palanisamy, Shuo Qie, Terrence A. Day, David Neskey, J. Alan Diehl, Viswanathan Palanisamy

In panel G of Fig 4, the specified hours (in the inset) for shControl-t_1/2_ and shFXR1-t_1/2_ are incorrectly switched. Please see the correct figure here.

**Fig 4:**
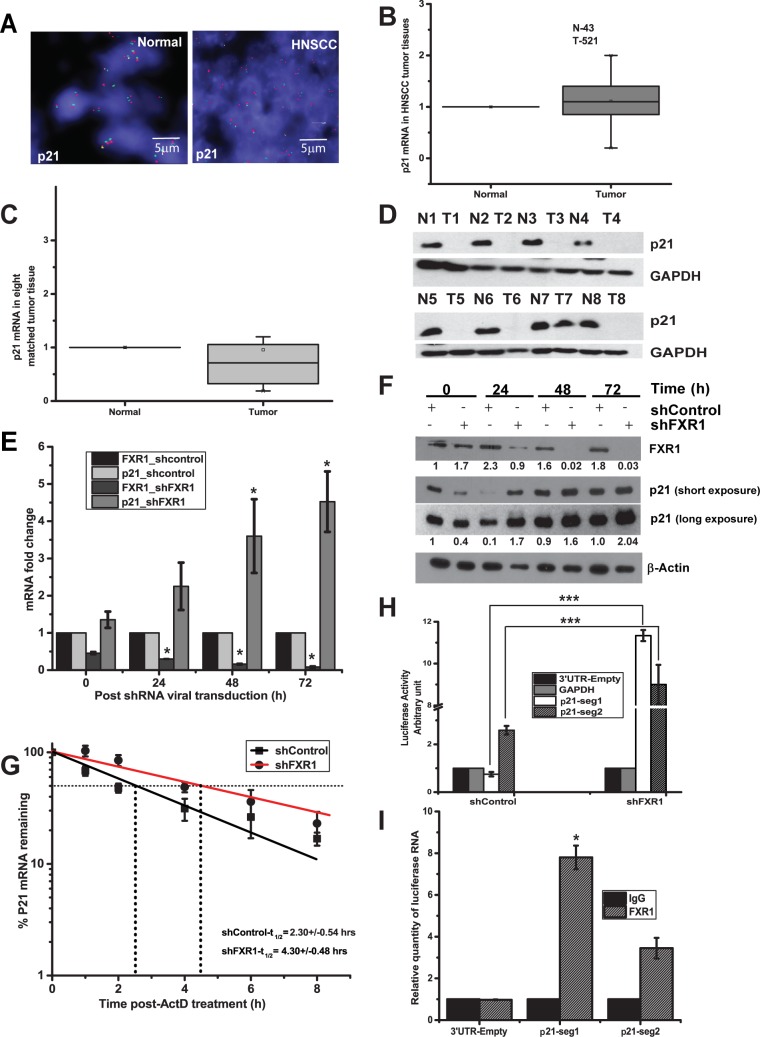
FXR1 destabilizes *p21* mRNA. (A) FISH analysis of p21 in a HNSCC TMA. Green indicates p21 and red denotes the control loci (scale bar 5μm). (B) Relative *p21* expression data obtained from cancer genome browser (N-43, T-521) (S4 Table). (C) Relative mRNA quantity of *p21* in eight matched HNSCC tumor compared to normal adjacent tissue samples estimated using qRT-PCR. GAPDH serves as a control. (D) Immunoblot analysis of p21 protein from eight representative matched HNSCC tumor and normal adjacent samples. GAPDH is used as a loading control. (E) Relative quantity of *FXR1* and *p21* levels estimated by qRT-PCR in UMSCC74B cells after treatment with FXR1 shRNA. Cells were collected at indicated time points. *FXR1* and *p21* levels in shControl treated UMSCC74B cells were taken as 1 for each time points. (F) Immunoblot analysis of p21 protein at different time points, as, mentioned in Fig 4E. (G) The mRNA decay rate of *p21* as indicated in UMSCC74B cells by qRT-PCR after silencing FXR1 followed by transcription inhibition with actinomycin-D for mentioned time points in the graph. Actin serves as a control. Data here represents the mean of n = 3 experiments. (H) Forty-eight hours after transfection of UM74B FXR1 KD and control cells with empty 3’UTR luciferase plasmid, luciferase-fused *GAPDH* 3′UTR plasmid or different segments of *P21* 3′UTR, the lysates were analyzed for luciferase activity using luminometer. The empty 3’UTR luciferase plasmid and luciferase-fused *GAPDH* 3′UTR served as a transfection and loading control. Values are the means ± SD from three independent experiments by using unpaired two sample t-test. (I) Binding of FXR1 with the 3′UTR of *p21seg1* and *p21seg2*RNAs at the G4 region. RNP IP was performed 48 h post-transfection of UMSCC74B cells with *seg1* and *seg2* 3′UTR fused to a luciferase reporter construct. Luciferase mRNA was detected using qRT-PCR. The luciferase gene in the empty-3′UTR was used as a transfection and qRT-PCR control. (*p<0.05, **p<0.005, ***p<0.0005).
