# Supplementary figures and images for: RNA-Binding Protein FXR1 Regulates p21 and TERC RNA to Bypass p53-Mediated Cellular Senescence in OSCC
Source: PLoS Genet. 2016 Sep 8;12(9):e1006306. doi: 10.1371/journal.pgen.1006306 (PMC5015924; doi:10.1371/journal.pgen.1006306)

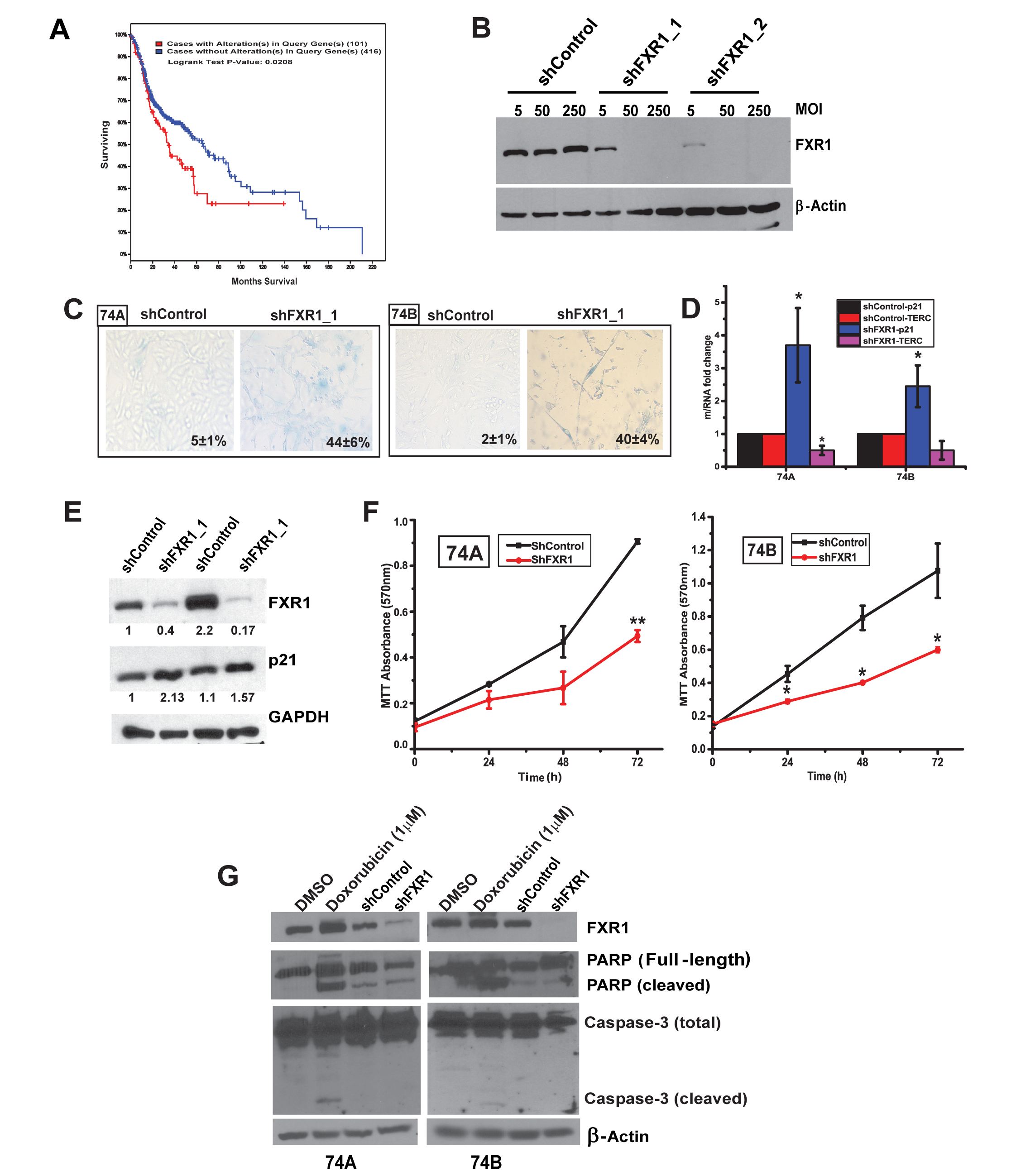

Supplement: S1 Fig — (A) Kaplan–Meier plots of overall survival of stage HNSCC patients (n = 522) stratified by FXR1 mRNA expression. The log-rank P values are shown. (B) Silencing of FXR1 in UMSCC74B cells. Cells were infected with lentivirus carrying short hairpin RNA (shRNA) against FXR1 in pLKO.1 hairpin vectors or with scrambled sequence containing vector used as control. Different multiplicity of infection (MOI) was used to titrate for the optimum knockdown. β-Actin was used as a loading control. (C) Staining for SA-β-gal in FXR1 depleted UMSCC74A and 74B cells with second shRNA directed to FXR1 (shFXR1_1 as shown in S1B Fig). (D) Relative quantity of p21 and TERC RNAs extracted from control and FXR1 KD cells (shFXR1_1) were estimated by using qRT-PCR. GAPDH serves as a control. (E) Immunoblot analysis of p21 protein in both FXR1 (shFXR1_1) depleted UMSCC74 and 74B cells. β-Actin was used as a loading control. (F) MTT analysis of cell viability in UMSCC74A and 74B cells transduced with control and FXR1 shRNA. Data presented as the mean ± SD of three experiments. (G) Western blots of FXR1 KD UMSCC74A and 74B cells for PARP and Caspase-3 cleavage. Apoptosis inducer for these cells, Doxorubicin was used to show relative PARP and Caspase-3 cleavage under drug induced apoptosis which was absent under FXR1 KD conditions. β-Actin was used as a loading control. (*p<0.05, **p<0.005, ***p<0.0005). (TIF) [file pgen.1006306.s001.tif]

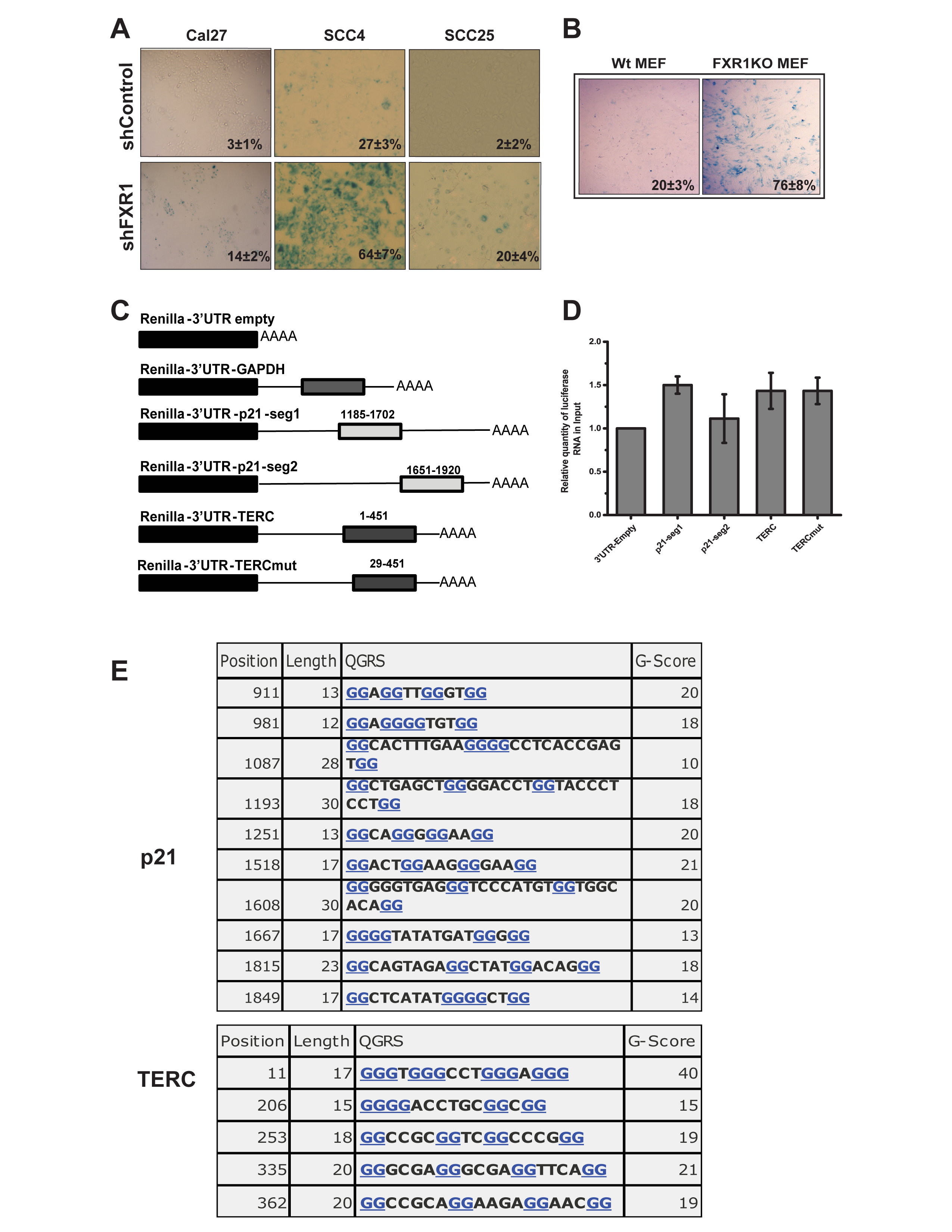

Supplement: S2 Fig — (A) SA-β-Gal staining of multiple oral cancer cells as indicated treated with shRNA control and shRNA FXR1. (B) Wild type and FXR1 KO Mouse embryonic fibroblasts (MEF) were stained with SA-β-gal for senescence associated β-galactosidase activity. (C) Schematic of luciferase 3’UTR constructs used in this study. Based upon the G-score (S2E Fig) the regions were carefully selected from p21 3’UTR and TERC RNA. (D) qRT-PCR analyses of luciferase RNA in the input samples used for RNP-IP analyses for high and low G4 RNA containing constructs. Empty-3’UTR plasmid and/or GAPDH serve as transfection and loading control, respectively (n = 2). (E) Two G4 structures containing RNAs, 3’-UTR of p21 and full-length TERC sequences were used for QGRS mapper software for determination of the G-score. Higher the G-score, stronger the G rich sequence that facilitates FXR1 binding. (TIF) [file pgen.1006306.s002.tif]

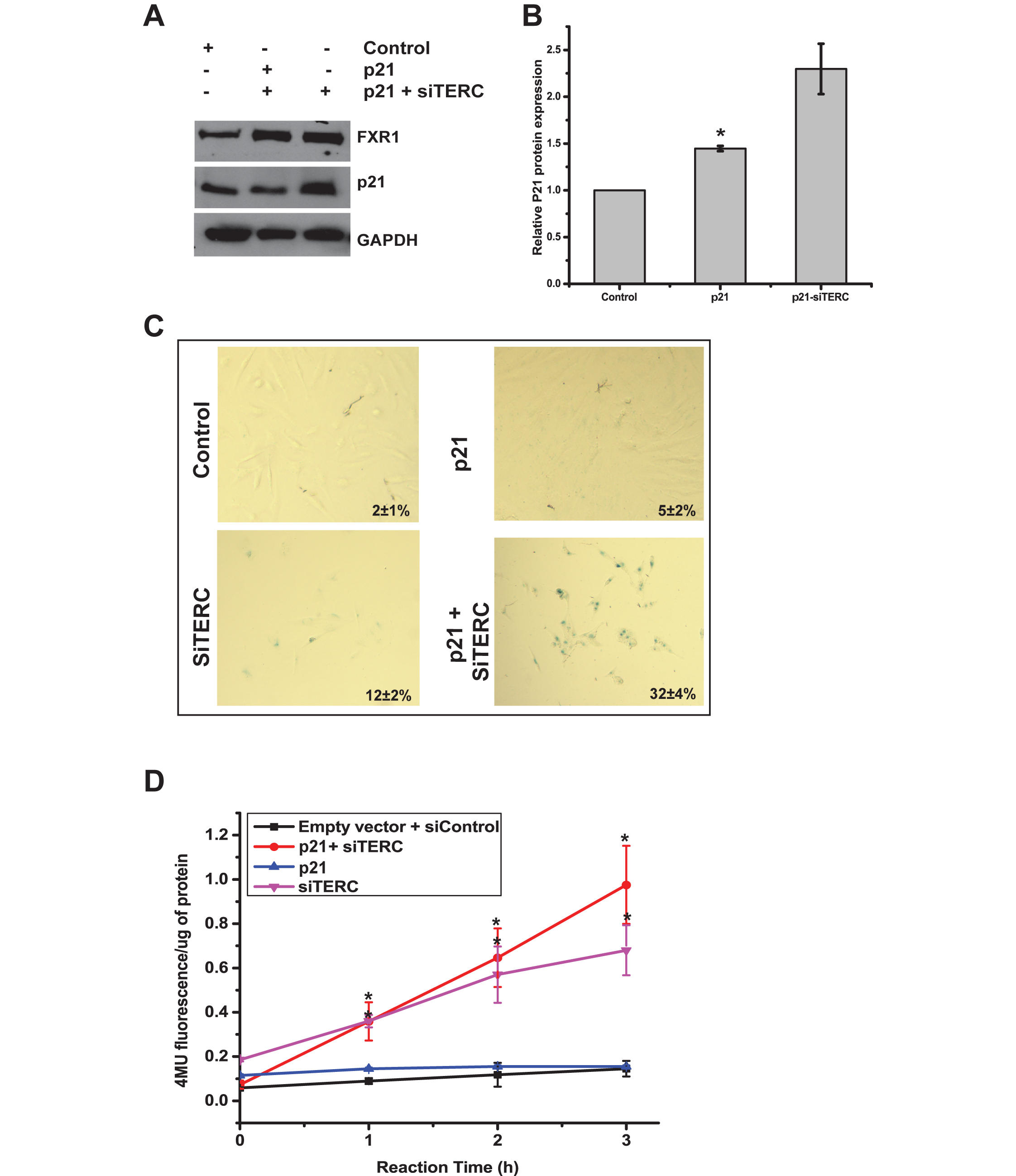

Supplement: S3 Fig — (A) Western blot to determine the protein change in UMSCC74A cells transfected independently or together with p21 overexpression plasmid or siTERC. (B) Quantification of p21 protein overexpression in 74A cells after transfection. (C) Expression of SA-β-gal activity in UMSCC74A cells transfected independently or together with p21 overexpression plasmid or siTERC RNA. (D) MUG conversion to 4-MU by senescence associated β-galactosidase was measured in these transfected cells. (*p<0.05, **p<0.005, ***p<0.0005). (TIF) [file pgen.1006306.s003.tif]
