# Supplementary material for: RNA-Binding Protein FXR1 Regulates p21 and TERC RNA to Bypass p53-Mediated Cellular Senescence in OSCC
Source: PLoS Genet. 2016 Sep 8;12(9):e1006306. doi: 10.1371/journal.pgen.1006306 (PMC5015924; doi:10.1371/journal.pgen.1006306)
Supplement: S1 Table — (DOCX) [file pgen.1006306.s004.docx]

| **RBP with DNA copy number variation and mutation (HNSCC 302)** | | | | |  |  |
| --- | --- | --- | --- | --- | --- | --- |
|  |  |  |  |  |  |  |
| **RRM domain** | | **Amplification>5%** |  | **Mutation** |  | **Deletion** |
| **PABPC1** |  | 5% |  |  |  |  |
| **IGF2BP2** |  | 18% |  |  |  |  |
| **TNRC6A** |  |  |  | 3.30% |  |  |
| **RBM3** |  |  |  |  |  | 5% |
| **ESRP1** |  | 5% |  |  |  |  |
| **ELAVL2** |  |  |  |  |  | 8% |
| **CPSF6** |  | 5% |  |  |  |  |
| **TRA2B** |  | 20% |  |  |  |  |
| **RBM26** |  |  |  | 3% |  |  |
| **U2SURP** |  | 12% |  |  |  |  |
| **PUF60** |  | 11% |  |  |  |  |
| **RBM4B** |  | 7% |  |  |  |  |
| **RBM4** |  | 8% |  |  |  |  |
| **ZRSR2** |  |  |  |  |  | 5% |
| **HNRNPA3** |  | 5% |  |  |  |  |
| **RBM14** |  | 7% |  |  |  |  |
|  |  |  |  |  |  |  |
| **KH- domain** | | **Amplification>5%** |  | **Mutation** |  | **Deletion** |
|  |  |  |  |  |  |  |
| **DDX53** |  |  |  |  |  | 5% |
| **KHDRBS3** |  | 11% |  |  |  |  |
| **ANKRD17** |  |  |  | 5% |  |  |
| **IGF2BP2** |  | 18% |  |  |  |  |
| **FXR1** |  | 21% |  |  |  |  |
| **ZC3H3** |  | 13% |  |  |  |  |
| **ZC3H15** |  | 6% |  |  |  |  |
| **MBNL1** |  | 15% |  |  |  |  |
| **CPSF4** |  | 6% |  |  |  |  |
| **ZC3H4** |  |  |  | 3% |  |  |
| **HELZ** |  |  |  | 4% |  |  |
| **TIPARP** |  | 15% |  |  |  |  |
| **LSM1** |  | 11% |  |  |  |  |
|  |  |  |  |  |  |  |
| **Cold-Shock Domain** | | **Amplification>5%** |  | **Mutation** |  | **Deletion** |
|  |  |  |  |  |  |  |
| **CSDE1** |  |  |  |  |  | 4% |
|  |  |  |  |  |  |  |
| **Ribsmal Prot S1-like** | | **Amplification>5%** |  | **Mutation** |  | **Deletion** |
|  |  |  |  |  |  |  |
| **TTC14** |  | 20% |  |  |  |  |
|  |  |  |  |  |  |  |
| **SURP module** | | **Amplification>5%** |  | **Mutation** |  | **Deletion** |
|  |  |  |  |  |  |  |
| **U2SURP/SR140** | | 12% |  |  |  |  |
|  |  |  |  |  |  |  |
| **YTH domain** | | **Amplification>5%** |  | **Mutation** |  | **Deletion** |
|  |  |  |  |  |  |  |
| **YTHDF3** |  | 5% |  |  |  |  |
|  |  |  |  |  |  |  |
| **Pumilio like Repeat** | | **Amplification>5%** |  | **Mutation** |  | **Deletion** |
|  |  |  |  |  |  |  |
| **KIAA0020** |  | 7% |  |  |  |  |
|  |  |  |  |  |  |  |
| **C2H2 Zn finger** | | **Amplification>5%** |  | **Mutation** |  | **Deletion** |
|  |  |  |  |  |  |  |
| **RBM10** |  |  |  |  |  | 5% |
| **ZFR** |  |  |  |  |  | 6% |
