# Supplementary material for: RNA-Binding Protein FXR1 Regulates p21 and TERC RNA to Bypass p53-Mediated Cellular Senescence in OSCC
Source: PLoS Genet. 2016 Sep 8;12(9):e1006306. doi: 10.1371/journal.pgen.1006306 (PMC5015924; doi:10.1371/journal.pgen.1006306)
Supplement: S2 Table — (DOCX) [file pgen.1006306.s005.docx]

| **RBP mRNA alteration, TCGA** | | |
| --- | --- | --- |
|  |  |  |
|  |  |  |
|  | **HNSCC** |  |
|  |  |  |
|  | **Up** | **Down** |
|  |  |  |
| U2AF2 | 8 | 4 |
| SPEN | 3 | 9 |
| PABPC1 | 15 | 0 |
| IGF2BP2 | 19 | 0 |
| RBM7 | 4 | 11 |
| CNOT4 | 5 | 12 |
| PPIE | 15 | 0 |
| RBM22 | 5 | 17 |
| TNRC6A | 5 | 6 |
| TRMT2A | 12 | 0 |
| POLDIP3 | 6 | 4 |
| SRSF5 | 10 | 0 |
| PABPN1 | 10 | 0 |
| PABPC1L | 13 | 0 |
| MTHFSD | 11 | 1 |
| ESRP1 | 9 | 1 |
| SNRNP70 | 13 | 0 |
| EIF3B | 19 | 0 |
| LARP4B | 13 | 5 |
| SRSF4 | 6 | 4 |
| PTBP3 | 8 | 4 |
| RBM18 | 9 | 1 |
| SLIRP | 11 | 0 |
| SRSF6 | 10 | 0 |
| SNRPB2 | 21 | 0 |
| RALY | 20 | 0 |
| RBM42 | 11 | 0 |
| HNRNPH2 | 9 | 2 |
| EIF3G | 12 | 1 |
| PPIL4 | 5 | 5 |
| RBM39 | 17 | 1 |
| RBM8A | 11 | 1 |
| GRSF1 | 7 | 5 |
| RBMX2 | 12 | 0 |
| SYNCRIP | 6 | 8 |
| TRA2B | 19 | 1 |
| SSB | 10 | 0 |
| G3BP2 | 8 | 4 |
| RBM26 | 8 | 4 |
| G3BP1 | 3 | 9 |
| TUT1 | 12 | 0 |
| CELF1 | 5 | 8 |
| SCAF4 | 5 | 6 |
| U2AF1L4 | 11 | 0 |
| U2SURP | 23 | 0 |
| ZRSR2 | 10 | 0 |
| HNRNPA3 | 10 | 0 |
| TAF15 | 11 | 1 |
| RBM4 | 16 | 0 |
| PUF60 | 45 | 0 |
| SRSF8 | 5 | 5 |
| ALYREF | 13 | 0 |
| RBM34 | 15 | 0 |
| RRP7A | 12 | 0 |
| NOL8 | 9 | 2 |
| SCAF8 | 10 | 3 |
| RBM12 | 6 | 5 |
| RBM17 | 9 | 2 |
| MCM3AP | 8 | 7 |
| PARP10 | 20 | 0 |
| KHSRP | 5 | 5 |
| FUBP3 | 9 | 9 |
| FXR1 | 24 | 0 |
| ANKRD17 | 8 | 9 |
| PNPT1 | 7 | 3 |
| HNRNPK | 7 | 8 |
| PCBP1 | 6 | 5 |
| MEX3C | 6 | 9 |
| ZC3H3 | 28 | 0 |
| RC3H2 | 5 | 10 |
| ZC3H11A | 9 | 3 |
| ZC3H15 | 7 | 3 |
| MKRN2 | 2 | 23 |
| RBM22 | 5 | 17 |
| RBM27 | 5 | 15 |
| ZC3H14 | 12 | 7 |
| ZC3H7B | 4 | 6 |
| TRMT1 | 13 | 0 |
| ZC3H13 | 5 | 20 |
| RNF113A | 12 | 0 |
| MKRN1 | 7 | 6 |
| NUPL2 | 13 | 0 |
| RBM26 | 6 | 6 |
| MBNL1 | 10 | 6 |
| ZC3H18 | 6 | 6 |
| U2AF1L4 | 11 | 0 |
| TIPARP | 14 | 0 |
| LENG9 | 11 | 0 |
| ZGPAT | 19 | 0 |
| HELZ | 5 | 6 |
| SNRPD3 | 14 | 0 |
| SNRPB | 17 | 0 |
| LSM4 | 12 | 0 |
| SNRPD1 | 12 | 0 |
| LSM1 | 17 | 2 |
| LSM10 | 11 | 0 |
| SNRPE | 16 | 0 |
| MCTS1 | 15 | 0 |
| DHX8 | 6 | 8 |
| SUPT6H | 4 | 6 |
| ZCCHC17 | 10 | 1 |
| EIF2S1 | 11 | 1 |
| PNPT1 | 9 | 1 |
| TTC14 | 18 | 0 |
| POLR2G | 21 | 0 |
| SF3A1 | 12 | 4 |
| SUGP1 | 12 | 1 |
| LARP1 | 2 | 10 |
| RBM25 | 7 | 3 |
| YTHDC2 | 7 | 7 |
| YTHDF1 | 19 | 2 |
| YTHDF3 | 15 | 5 |
| THUMPD1 | 6 | 5 |
| THUMPD2 | 10 | 0 |
| THUMPD3 | 9 | 10 |
| PUM2 | 5 | 5 |
| KIAA0020 | 20 | 2 |
| PUM1 | 3 | 12 |
| SAMD4B | 9 | 6 |
| GTF3A | 7 | 3 |
| ZFR | 10 | 6 |
| KIN | 6 | 5 |
| TROVE2 | 7 | 3 |
