# Supplementary material for: RNA-Binding Protein FXR1 Regulates p21 and TERC RNA to Bypass p53-Mediated Cellular Senescence in OSCC
Source: PLoS Genet. 2016 Sep 8;12(9):e1006306. doi: 10.1371/journal.pgen.1006306 (PMC5015924; doi:10.1371/journal.pgen.1006306)
Supplement: S6 Table — (DOCX) [file pgen.1006306.s009.docx]

**Table S6 List of Oligonucleotide sequences**

| **Oligonucleotide** | **Sequence** |
| --- | --- |
| FXR1-F | CCCTAATTACACCTCCGGTTATG |
| FXR1-R | TCTCCTGCCAATGACCAATC |
| FMR1-F | GGTCGAGGTAGTAGACCTTACA |
| FMR1-R | GTTCGTCTCTGTGGTCAGATTC |
| FXR2-F | GGTTCGAGTGGAAGGTGATAAT |
| FXR2-R | GAGAGGTGATACTCCAGCAAAG |
| CDKN1A-F | CGGAACAAGGAGTCAGACATT |
| CDKN1A-R | AGTGCCAGGAAAGACAACTAC |
| CDKN1B-F | CTAACTCTGAGGACACGCATTT |
| CDKN1B-R | TGCAGGTCGCTTCCTTATTC |
| P53-F | CACTCTTGCCCACCCATAAA |
| P53-R | GAACTCCTGACCTTGTGATCTG |
| PTEN-F | CCCACCACAGCTAGAACTTATC |
| PTEN-R | TCGTCCCTTTCCAGCTTTAC |
| TERC-F | TTTGTCTAACCCTAACTGAGAAGG |
| TERC-R | CTCTAGAATGAACGGTGGAAGG |
| GAPDH-F | GGTGGTCTCCTCTGACTTCAACA |
| GAPDH-R | GTTGCTGTAGCCAAATTCGTTGT |
| pLS_3'UTR-p21seg1-F | CTAATCTAGAGCTAGCGGTTATCTCTGTGTTAGG |
| pLS_3'UTR-p21seg1-R | GCGGCCGGCCCTCGAGAAGCACTTCAGTGCCTCC |
| pLS_3'UTR-p21seg2-F | CTAATCTAGAGCTAGCCAGCCTAGGGCTGAGCTG |
| pLS_3'UTR-p21seg2-R | GCGGCCGGCCCTCGAGCCTCCTAGAAAGATCTAC |
| pLS_3'UTR-TERC-F | CTAATCTAGAGCTAGCGGGTTGCGGAGGGTGGGC |
| pLS_3'UTR-TERC-R | GCGGCCGGCCCTCGAGGCATGTGTGAGCCGAGTC |
| pLS_3'UTR-TERCmut-F | CTAATCTAGAGCTAGCGGGTGGTGGCCATTTTTT |
| Luciferase-F | AGATCATCTTTGTGGGCCACGACT |
| Luciferase-R | TCTCAGCATGCACGATAGCCTTGA |
